# Low-grade inflammation is associated with a heterogeneous lipoprotein subclass profile in an apparently healthy population sample

**DOI:** 10.1186/s12944-023-01856-6

**Published:** 2023-07-11

**Authors:** Daniel L. Pontzen, Martin Bahls, Diana Albrecht, Stephan B. Felix, Marcus Dörr, Till Ittermann, Matthias Nauck, Nele Friedrich

**Affiliations:** 1 University Medicine Greifswald, Ferdinand-Sauerbruch-Str. 17475, Greifswald, Germany; 2 German Centre for Cardiovascular Research (DZHK), Partner Site Greifswald, Greifswald, Germany; 3 Institute for Community Medicine, SHIP-KEF, University Medicine Greifswald, Greifswald, Germany; 4 Leibniz Institute Greifswald, Leibniz Institute for Plasma Science and Technology eV, Greifswald, Germany; 5 Institute of Clinical Chemistry and Laboratory Medicine, University Medicine Greifswald, Greifswald, Germany

**Keywords:** Lipidomics, Inflammation, Epidemiology, Prevention of cardiovascular diseases, Prevention of metabolic diseases, Chronic non-communicable diseases, Cytokines, Inflammatory biomarkers, Dyslipidemia, Cardiovascular disease, Nuclear magnetic resonance spectroscopy, Lipoprotein particles, Lipoprotein subclasses, Immunometabolism, Soluble BAFF, Soluble APRIL, MMP2, Gelatinase A, TNF, Heart-liver axis, Lipid metabolism, VLDL, LDL, HDL, Lipolysis, Energy mobilization, Residual risk, Apolipoprotein concentration, Small dense LDL

## Abstract

**Background and aims:**

Prevention measures for cardiovascular diseases (CVD) have shifted their focus from lipoproteins to the immune system. However, low-grade inflammation and dyslipidemia are tightly entangled. The objective of this study was to assess the relations between a broad panel of inflammatory biomarkers and lipoprotein subclass parameters.

**Methods:**

We utilized data from the population-based Study of Health in Pomerania (SHIP-TREND, *n* = 403). Plasma concentrations of 37 inflammatory markers were measured by a bead-based assay. Furthermore, we employed nuclear magnetic resonance spectroscopy to measure total cholesterol, total triglycerides, total phospholipids as well as the fractional concentrations of cholesterol, triglycerides, phospholipids, ApoA1, ApoA2 and ApoB in all major lipoprotein subclasses. Associations between inflammatory biomarkers and lipoprotein subclasses were analyzed by adjusted linear regression models.

**Results:**

APRIL, BAFF, TWEAK, sCD30, Pentraxin-3, sTNFR1, sTNFR2, Osteocalcin, Chitinase 3-like 1, IFN-alpha2, IFN-gamma, IL-11, IL-12p40, IL-29, IL-32, IL-35, TSLP, MMP1 and MMP2 were related with lipoprotein subclass components, forming two distinct clusters. APRIL had inverse relations to HDL-C (total and subclasses) and HDL Apo-A1 and Apo-A2 content. MMP-2 was inversely related to VLDL-C (total and subclasses), IDL-C as well as LDL5/6-C and VLDL-TG, IDL-TG, total triglycerides as well as LDL5/5-TG and HDL4-TG. Additionally, we identified a cluster of cytokines linked to the Th1-immune response, which were associated with an atherogenic lipoprotein profile.

**Conclusion:**

Our findings expand the existing knowledge of inflammation-lipoprotein interactions, many of which are suggested to be involved in the pathogeneses of chronic non-communicable diseases. The results of our study support the use of immunomodulatory substances for the treatment and possibly prevention of CVD.

**Supplementary Information:**

The online version contains supplementary material available at 10.1186/s12944-023-01856-6.

## Introduction

Despite significant advancements in the treatment and prevention of cardiovascular diseases (CVD) over the last decades, CVD remain the leading cause of morbidity and mortality in Western societies [1]. The development and progression of CVD is strongly associated with an atherogenic lipoprotein profile. High-density lipoprotein cholesterol (HDL-C) and low-density lipoprotein cholesterol (LDL-C), together with other components of the Framingham risk score, predict approximately 75% of CVD risk [2]. Plasma lipid biomarkers HDL-C, LDL-C and total triglycerides (TG) have been used to assess CVD risk for decades [3, 4]. However, even with modern, highly efficient LDL-C lowering therapies, patients are still at significant risk to suffer from CVD incidents [5]. Thus, current research focuses on investigating the remaining residual risk. Non-standard lipoprotein parameters, including particle number, size distribution and apolipoprotein concentration could be such targets. In particular, increases of apolipoprotein B (apoB) carrying particles and a predominance of small LDL particles are independently associated with CVD progression und incidence [6]. Overall, lipids remain a hallmark for preventive cardiology.

In addition, the importance of chronic and metabolic inflammation plays a key role in the pathogenesis of cardiometabolic diseases. Previous studies have demonstrated that the innate immune system may be instrumental in slowing disease progression and enhancing healing capacities [7, 8]. The lipid metabolism and the immune system are tightly entangled with numerous bidirectional dependencies. Infection and subsequent immune responses such as inflammation, fever, acute-phase reaction and tachycardia are highly energy consuming processes s. In addition, chronic and metabolic inflammation plays a key role in the pathogenesis of cardiometabolic diseases [9].

The present study aimed to further explore the relations between inflammation and parameters of immune activation with lipoprotein subclasses. An improved understanding of the inflammation-lipoprotein interaction may provide insights into maladaptive processes that are involved in the pathogeneses of most non-communicable chronic inflammatory diseases like CVD.

## Patients and methods

### Study population

SHIP-TREND is the second cohort of the Study of Health in Pomerania (SHIP), a population-based research project in West Pomerania, a rural region in north-east Germany [10]. A stratified random sample of 8,826 adults aged 20–79 years was drawn from population registries. Sample selection was facilitated by centralization of local population registries in the Federal State of Mecklenburg-West Pomerania. Stratification variables were age, sex and city/county of residence. Out of all invitations, 4,420 individuals chose to participate (50.1% response) in the examinations between 2008 and 2012. The study followed the recommendations of the Declaration of Helsinki and was approved by the ethics committee of the University of Greifswald. SHIP data is publicly available for scientific and quality control purposes. Data usage can be applied for via www.community-medicine.de.

In 503 SHIP-TREND-0 participants without self-reported diabetes mellitus, a panel of inflammatory markers were measured (see measurements). Of these participants, 97 study participants were excluded due to the presence of at least one of the following conditions (overlap might exist): no nuclear magnetic resonance (NMR) spectroscopy (*n* = 10), estimated glomerular filtration rate (eGFR) < 30 mL/min/1.73 m^2^ (*n* = 2), left ventricular ejection fraction < 40% (*n* = 1), history of cancer (*n* = 19), intake of anti-inflammatory and antirheumatic products (anatomic-therapeutic-chemical [ATC] code M01) or lipid modifying agents (ATC C10) (*n* = 61) or missing values for confounding factors (*n* = 4). Consequently, the final study population comprised 403 individuals.

### Measurements

Participants’ characteristics and medical histories were recorded using computer-aided personal interviews. Smoking status was categorized as current, former or never smoker. Mean daily alcohol consumption was calculated based on volume proportions of pure ethanol in specific beverages. Diabetes mellitus was defined through self-reporting, where participants were asked whether they had ever been diagnosed with diabetes mellitus by a physician or if they were taking antidiabetic medication (ATC A10). Height was measured to the nearest 1 cm using a digital ultrasound instrument, while weight was measured using standard digital scales to the nearest 0.1 kg. Waist circumference was measured at the narrowest part of the waist in comfortably standing participants using an inelastic tape with equidistance between the lower rib margin and the iliac crest. The measurements were then rounded to the nearest 0.1 cm. All measurements were taken with the participants wearing light clothing and without shoes. Body mass index (BMI) was calculated as weight (kg) / height^2^ (m^2^). All participants were offered a bioelectrical body impedance analysis (BIA). BIA analyses were performed using a Nutriguard M device and NutriPlus software (Data Input GmbH, Darmstadt, Germany). Charges of 5 kHz, 50 kHz and 100 kHz were applied to measure resistance, reactance and phase angle. Lean body mass was automatically calculated from the latter measurements within the NutriPlus software (Data Input GmbH, Darmstadt, Germany). After a 5-min resting period, blood pressure was measured three times on seated participants using a digital BP monitor (HEM-705CP, Omron, Japan) with a further 3-min resting period between readings. The mean of the second and third measurements was used to calculate the systolic and diastolic blood pressure. Echocardiography was performed by certified physicians in two-dimensional and M-mode using a Vingmed CFM 800A system (GE Medical Systems, Waukesha, Wisconsin, USA). M-mode images of the left ventricle were recorded mid-papillary level in the parasternal short-axis view. Left ventricular proportions, including interventricular septum thickness (IVS), posterior wall thickness (LVPW), left ventricular end-diastolic diameter (LVDD) and left ventricular end-systolic diameter (LVDS), were measured using the leading-edge technique. Left ventricular mass index (LVMI) was calculated based on the method described by Devereux et al. and then index to height using the formula: LVMI = (0.80 × (1.04 × ((LVDD + IVS + LVPW)3—LVDD3)) + 0.60)/height^2.7 [11]. Left ventricular endocardial fractional shortening (LVEFS) was calculated according to the guidelines of the American Society of Echocardiography [12]. The assessment of intra-reader, intra-observer, inter-reader, and inter-observer variability for all echocardiographic measurements resulted in Spearman correlation coefficients exceeding 0.85, indicating strong agreement. The mean differences (± 2 standard deviations) between measurements were found to be less than 5% [13]. All data and measurements were stored digitally.

### Laboratory methods

Blood samples of overnight fasted participants were taken from the cubital vein in the supine position between 7 a.m. and 1 p.m. Serum and plasma samples were stored at − 80 °C in the Integrated Research Biobank (Liconic, Lichtenstein) of the University Medicine Greifswald and used in accordance with its regulations. Total cholesterol, HDL-C, LDL-C, total triglycerides and creatinine were measured using the Dimension Vista 500 analytical system (Siemens AG, Erlangen, Germany) as well as with NMR (see below) except creatinine. Aspartate amino transferase (ASAT), alanine amino transferase (ALAT) and gamma-glutamyl transferase (GGT) concentrations were determined photometrically (Hitachi 704; Roche, Mannheim, Germany). Glycated hemoglobin (HbA1c) was measured by high-performance liquid chromatography (Diamat, Bio-Rad Laboratories, Munich, Germany). The estimated glomerular filtration rate (eGFR) was calculated using the CKD-EPI equation [14].

A total of 37 inflammatory biomarkers (IBM) were measured in EDTA plasma using a bead-based assay (Bio-Plex Pro™ Human Inflammation Assay Panel 1, Bio-Rad Laboratories, Hercules, CA). In short, 50 μl of the coupled magnetic bead mixture were added to each well in a 96-well plate, along with serial dilutions of the reconstituted standard, blanks, controls and the participants plasma samples (1:4 diluted). This mixture was incubated in the dark at room temperature (shaking at 850 rpm) over night and the plates were subsequently washed three times. The detection antibody mixture was then added to the wells for an additional 30 min incubation step at room temperature (shaking at 850 rpm), followed by washing three times and adding streptavidin-PE for 10 min at room temperature (shaking at 850 rpm). The samples were washed three times and the plates were measured on a FLEXMAP3D® (Luminex Corp.) machine using xPONENT® v4.2 software. The quantitative analysis was performed using a 5P-logistic regression model on the standard dilution curve data to calculate absolute quantitative data of the study samples (xPONENT® v4.2 software). After quality control, six parameters were excluded from the analyses. A median coefficient of variation of 7.7% was achieved. The coefficient of variations for all considered parameters are given in Table S1. As measurements were done in six batches, we corrected for differences between the plates using a median normalization.

### ^*1*^*H-NMR—lipoprotein subclass analysis*

Plasma samples were stored frozen at -80 °C until analysis. After thawing, 250 μL of plasma were mixed with 250 μL of phosphate buffer [prepared with D2O and contained sodium 3-trimethylsilyl-(2,2,3,3-D4)-1-propionate (TSP) as reference, (pH 7.4)]. Spectra were recorded on a Bruker AVANCE-II 600 NMR spectrometer operated by TOPSPIN 3.2 software (both Bruker Biospin, Rheinstetten, Germany), equipped with 5-mm z-gradient probe (Bruker Biospin, Rheinstetten, Germany) and automated tuning and matching (ATMA) unit (Bruker Biospin, Rheinstetten, Germany). Specimens were automatically delivered to the spectrometer via SampleJet (Bruker Biospin, Rheinstetten, Germany) into standard 5 mm NMR tubes. The acquisition temperature was set to 310°K. A standard one-dimensional ^1^H-NMR pulse sequence with suppression of the water peak (NOESYPREAST) was used. The sequence has the form –RD-gz,1–90°-t-90°-tm-gz,2-ACQ, where RD is the relaxation delay (4 s) t is a short delay (~ 3 µsec), 90° represents the 90° RF hard pulse, tm is the mixing time (10 ms), gz,1 and gz,2 are the magnetic field z gradients both applied for 1 ms and ACQ is the acquisition period (2.7 s) collecting 98,304 data points at a sweep width of 30 ppm. The receiver gain is set at 90.5 for all experiments. For pre-processing, a line broadening of 0.3 Hz, a zero filling to produce 128 k data points and a first-order phase correction of 0.0 was applied. Spectral processing included zerofilling, linebroadening, Fourier transformation and referencing of the chemical shift and determination of the spectral intensity per 1 mmol protons for quantitative referencing. Chemical shifts of plasma spectra were referenced to the CH3-group signal of alanine adjusting it to 1.48 ppm. Spectra were submitted to data analysis for lipoprotein subclass and apolipoprotein analysis B.I.LISA™ (Bruker BioSpin GmbH Germany). All laboratory methods were performed by skilled technical personnel and according to manufacturers’ recommendations.

The clinically most established lipid parameters (total cholesterol, total triglycerides, LDL-C and HDL-C) measured by NMR were compared with the standard laboratory measurements (Dimension Vista 500) using Passing-Bablok regression and Pearson correlation (see supplementary Figure S1). Good agreement was archive for all four parameters (Pearson correlation coefficient *r* > 0.90 for all 4 comparisons).

### Statistical analyses

Nominal data were shown as a percentage (N) and continuous data are expressed as median (25% quartile; 75% quartile). Based on Pearson correlation coefficients, a correlation matrix for the considered inflammation parameters was calculated. Linear regression models were performed to determine the associations between inflammatory markers (exposure, standardized by the SD) and lipoprotein subclasses (outcomes, standardized by the SD). Models were adjusted for age, sex, smoking and lean body mass. To account for multiple testing, we adjusted the *p* values from regression analyses by controlling the false discovery rate (FDR) at 5% using the Benjamini–Hochberg procedure. To identify similar association patterns in lipoprotein subclasses as well as inflammatory markers hierarchical clustering were performed based on the results of the linear regressions. In sensitivity analyses, waist circumference or the fatty liver index was used as confounder instead of lean body mass. FLI was calculated according to the formula published by Bedogni et al. [15]. Furthermore, interaction terms for lean body mass, waist circumference or fatty liver index and cytokines were included in the models. After adjustment for multiple testing, no interaction term was significant. Statistical analyses were performed using SAS 9.4 (SAS Institute Inc., Cary, North Carolina, USA).

## Results

### Study population

The general characteristics of the study population are provided in Table 1. Of the participants, 47.5% were men, with a mean age of 48.1 years and 24% were current smokers. Physical examination revealed a mean lean body mass of 56.3 kg with a mean BMI of 27.2 kg/m^2^ and mean systolic or diastolic blood pressure values of 123.7 mmHg or 76.6 mmHg, respectively. Mean eGFR was 98.5 ml/min/1.73m^2^. Participants reported an average daily alcohol consumption equivalent to 8.7 g/day of pure ethanol. Additional population characteristics, especially regarding the considered inflammatory marker concentrations are displayed in supplementary Table S1. Total cholesterol, total triglycerides, LDL-C and HDL-C levels measured by NMR spectroscopy were, 253 mg/dl, 125 mg/dl, 152 mg/dl and 66 mg/dl respectively.

**Table 1:** General characteristics of the study population

	**Study population** **(*N* = 406)**
Men, %	47.5 (193)
Age, years	48 (39; 58)
Smoking, %	
Never smoker	41.4 (168)
Ex-smoker	34.2 (307)
Current smoker	24.4 (99)
Alcohol consumption, g/d	4.9 (1.5; 10.9)
BMI, kg/m^2^	26.7 (24.1; 30.0)
Lean body mass, kg	53.3 (46.8; 65.4)
Waist circumference, cm	87 (79; 97)
HbA1c, %	5.1 (4.8; 5.5)
Systolic BP, mmHg	123.5 (112.0; 134.0)
Diastolic BP, mmHg	76.0 (70.0; 82.5)
LVMI, g/m^2.7^	39.1 (31.5; 47.8)
Fractional shortening, %	42.0 (37.1; 47.5)
History of myocardial infarction, %	0 (0)
History of stroke, %	0.7 (3)
History of heart failure, %	2.0 (8)
eGFR, ml/min/1.73m^2^	99.0 (89.7; 108.6)
ASAT, µkatal/l	0.30 (0.14)
ALAT, µkatal/l	0.43 (0.25)
GGT, µkatal/l	0.63 (0.55)
Total cholesterol, mg/dl^a^	252 (219; 283)
LDL cholesterol, mg/dl^a^	148 (126; 175)
HDL cholesterol, mg/dl^a^	64 (56; 76)
Triglycerides, mg/dl^a^	109 (74; 154)

*BMI* Body mass index, *BP* Blood pressure, *eGFR* Estimated glomerular filtration rate, *HbA1c* Glycated hemoglobin, *LVMI* Left ventricular mass index, *HDL* High-density lipoprotein, *LDL* Low-density lipoprotein, *ALAT* Alanine aminotransferase, *ASAT* Aspartate aminotransferase, *GGT* Gamma-glutamyltransferase. Continuous data are expressed as median (Q1; Q3); Nominal data are given as percentages (N). ^a^Measured by NMR spectroscopy

### Lipoprotein subclasses

To determine whether changes in lipid concentrations were the result of raised particle number or a compositional shift, we performed a within-group correlation analysis as shown in supplementary Figure S2. We found strong correlations (with Pearson correlation coefficients > 0.9, except for HDL4) between cholesterol and phospholipids content across all subclasses of LDL, VLDL, and HDL particles. Additionally, in VLDL (but not LDL and HDL particles), both cholesterol and the phospholipids content were strongly correlated to triglyceride content, indicating a highly consistent lipid composition across different VLDL particle diameters (Figure S2). The triglyceride content of LDL and HDL subclasses, on the other hand, showed a greater degree of variance. Based on the strong positive correlations between cholesterol and the phospholipids content in all considered particles, we decided to omit phospholipids contents from further analyses. Likewise, VLDL-TG were not further considered. In total, 59 lipoprotein parameters were considered.

### Inflammatory biomarkers

**Fig. 1:**
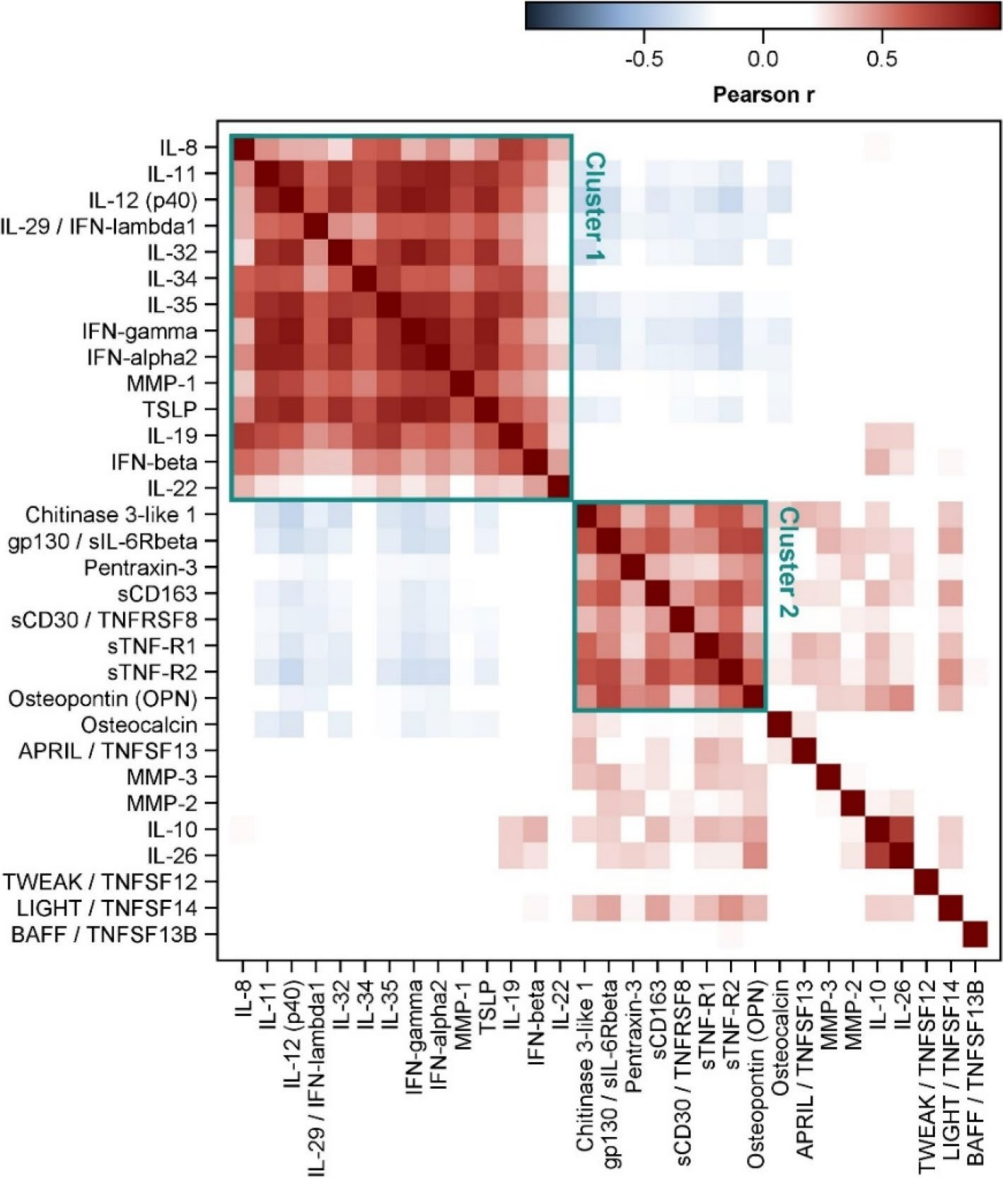
Color-coded Pearson correlation coefficients for the considered inflammatory markers. The exact coefficients for the two selected clusters are displayed in Figure S3

Correlations between the measured inflammatory markers were calculated and visualized (Fig. 1 and S3). Two clusters, Cluster 1 and Cluster 2, including strongly positive correlated inflammatory markers were identified. Cluster 1 included IL-8, IL-11, IL-12p40, IL-19, IL-22, IL-29, IL-32, IL-34, IL-35, IFN-alpha2, IFN-beta, IFN-gamma, MMP-1 and TSLP. Cluster 2 comprised Osteopontin, Pentraxin-3, Chitinase-3-linke-1, gp130, sTNF-R1, sTNF-R2 and sCD30. Whereas the markers within these two clusters positively correlated with a Pearson coefficient up to *r* = 0.91, we observed inverse correlations between these two clusters. However, these relations were much weaker.

### Association of 1H-NMR-based lipoprotein subclasses and inflammatory markers

**Fig. 2:**
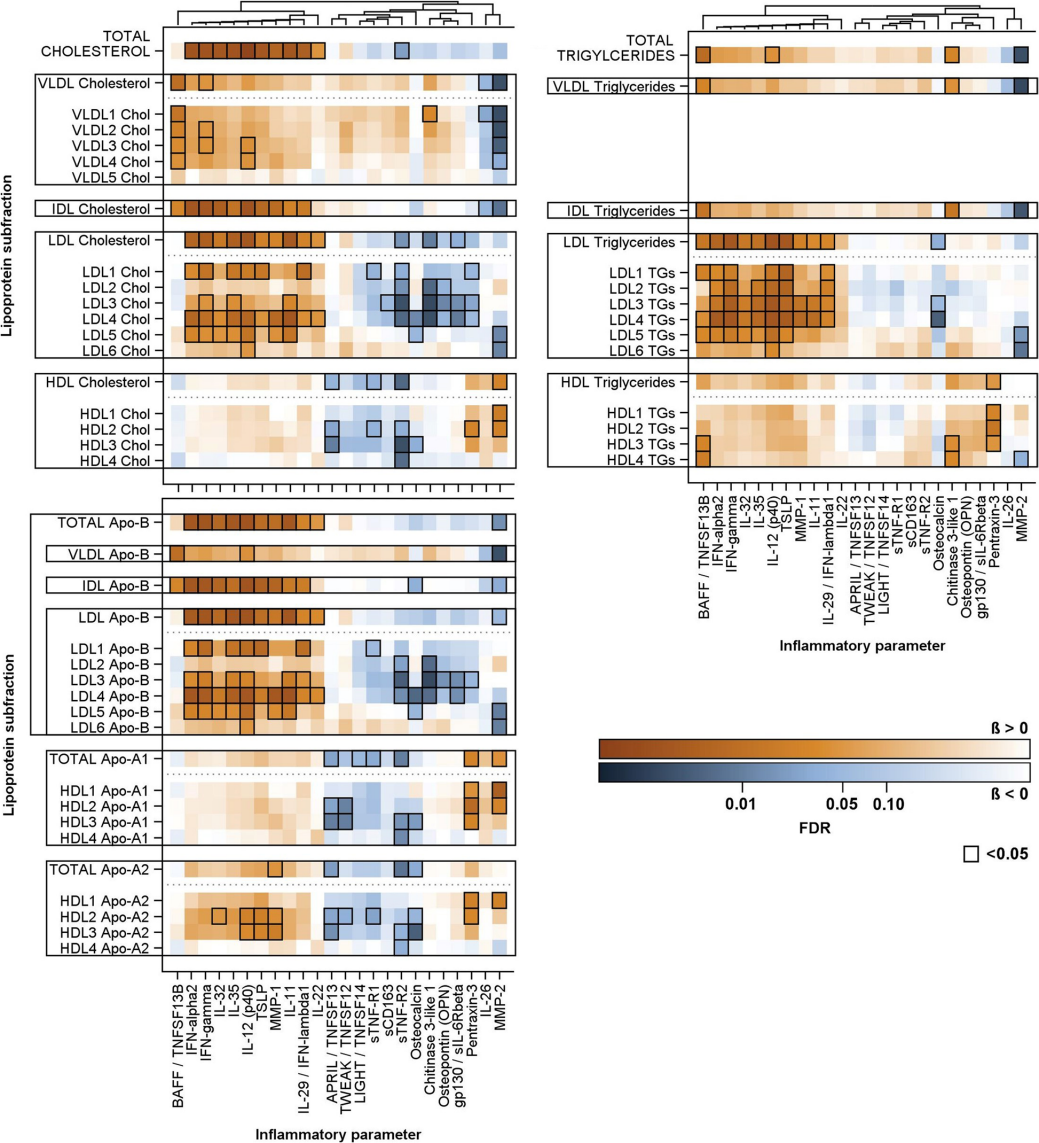
Color coded corrected *P* values (controlling the false discovery rate (FDR) at 0.05) from linear regression analyses for the associations of lipoprotein subclasses with inflammatory parameters. Significant associations (FDR < 0.05) are marked with a black box. All analyses were adjusted for age, sex, smoking behavior (never, ex or current) and lean body mass. Orange and blue shading indicate positive and inverse associations, respectively. The full matrix with all investigated associations is given in Figure S4

After adjusting for age, sex, smoking and lean body mass, linear regression models revealed a broad range of associations between lipoprotein subclasses and inflammatory markers (Fig. 2 and S4, Table S2), demonstrating clear association patterns linked to the identified inflammatory correlation clusters. Several sensitivity analysis did not alter the results of the main analysis. Most inflammatory markers in cluster 1 including IL-11, IL-29, IL-32, IL-35, IL-12p40, MMP-1, TSLP, IFN-alpha2 and IFN-gamma showed positive associations with total cholesterol and cholesterol content in IDL and LDL (total and subclasses) particles. With respect to triglycerides, these associations were also reflected in positive associations to total LDL-TG and LDL-TG in most subclasses. However, among the Cluster 1 inflammatory markers, only IL-12p40 exhibited significant associations to total triglycerides, while no such associations existed with IDL-TG. The LDL-Apo-B, IDL-Apo-B and total-Apo-B showed, as expected, the same pattern as the cholesterol content in these classes. The soluble TNF ligand superfamily member 13B (also known as B-cell-activating factor of the TNF family [BAFF]) displayed a different association pattern. While positive associations with LDL-TG (total and LDL1/4/5) were observed similar to the cluster 1 inflammatory markers, additional relations to the IDL-TG, VLDL-TG and total triglycerides were seen. With respect to cholesterol content, in contrast to the cluster 1, positive association were observed to IDL-C and VLDL-C (subclasses and total) but not to LDL-C and total cholesterol. Interestingly, the soluble TNF ligand superfamily member 13 (also known as a proliferation-inducing ligand [APRIL]) was not related to total triglycerides. At the same time, it showed inverse relations to HDL-C (total and subclasses) and HDL Apo-A1 and Apo-A2 content. Beside Cluster 1 markers, inverse associations of Cluster 2 markers including STNF-R2, Chitinase 3-like 1, gp130 and osteopontin were observed with LDL-cholesterol (total and/or subclasses) but not LDL-TG (total and subclasses). Of these, sTNF-R2 was also strongly inversely related to HDL-C (total and subclasses) and HDL Apo-A1 and Apo-A2 content. A distinct association pattern was shown by MMP-2 (Fig. 2) which levels were strongly inversely related to VLDL-C (total and subclasses), IDL-C as well as LDL5/6-C and VLDL-TG, IDL-TG, total triglycerides as well as LDL5/5-TG and HDL4-TG. Additionally, MMP-2 was associated with HDL-C (total HDL and HDL1/2), Apo1 (total and HDL1/2) and HDL1 Apo-A2.

## Discussion

The present study examined the relations between makers of inflammation and different lipoprotein subclasses in an apparently healthy subgroup of a population-based cohort. Our main findings provide novel evidence for two potential interactive pathways supporting the heightened energy demands during systemic inflammation. First, we observed an innovative link between the BAFF/APRIL system and the lipid metabolism supporting a proposed role in mobilization of energy during an acute or chronic immune response. Secondly, we provide new evidence supporting a relatively unexplored MMP2 heart-liver-axis, by which the heart can mobilize energy-rich triglycerides from hepatic storage. Lastly, we provide further insight into the impact of a broad immune response on the systemic lipoprotein metabolism.

### The BAFF/APRIL system demands and supplies

The TNF superfamily members BAFF and APRIL are vital for B cell proliferation, maturation, function and survival [16]. They are released into the circulation in response to inflammatory signals like IFN-gamma or IFN-beta. This secretion of BAFF/APRIL is triggered by pathogen-associated molecular patterns (PAMPs) or damage-associated molecular patterns (DAMPs) via pattern recognition receptors (PRRs) including lectin-like oxidized LDL receptor-1 (LOX-1) or various toll-like receptors (TLRs) [17]. BAFF and APRIL are also involved in the development of atherosclerosis with subsequent CVDs [18]. More recently, several reports implicated BAFF and APRIL in insulin resistance, lipolysis, brown adipose tissue dependent thermogenesis and non-alcoholic fatty liver disease, suggesting a broader role of these cytokines with regards to energy metabolism [19, 20]. The present study reports significant associations of BAFF/APRIL and plasma lipids in an apparently healthy human cohort. In short, BAFF was associated with VLDL-ApoB, -C, -PL and -TG in with an emphasis on larger VLDL particles, IDL-ApoB, -C, -PL, -TG and triglycerides in LDL1, LDL4, LDL5, HDL3 and HDL4. Meanwhile APRIL was inversely associated with HDL-ApoA1, ApoA2, -cholesterol and -phospholipids in general and in medium sized HDL2 and HDL3, while the larger and smaller HDL1 and HDL4 did not show such associations. To our knowledge, significant associations between BAFF/APRIL and plasma lipids have only been reported in small sample of patients with membranous nephropathy [21], children with ANCA-associated glomerulonephritis (GN) [22] and in patients with obesity [23]. Both BAFF and APRIL induce lipolysis in white adipocytes, as indicated by elevated expression of adipose triglyceride lipase and hormone-sensitive lipase and free glycerol supernatant [24]. This process seems to be independent from immune cell mediation and produces free fatty acids and glycerol which then diffuse into the plasma. From there they are transported to the liver and other tissues [24]. Studies on BAFF knockout mice showed significantly lower hepatic triglyceride and cholesterol levels indicating a lower fatty acid influx and lipogenesis during BAFF deficiency [25]. Increased triglyceride supply is considered a strong trigger of VLDL production [26]. Additionally, in vitro research in 3T3-L1 adipocytes showed decreased levels of lipoprotein lipase mRNA expression after treatment with BAFF, indicating than BAFF might also slow the plasmatic clearance of VLDLs [27]. Taken together these pathways provide mechanistic insight into the strong associations between BAFF and VLDL we observed.

The elevated levels of IDL-ApoB, -C, -PL, and -TG as well and triglycerides in LDL1, LDL5 and LDL6 particles we observed are products of VLDL after delipidation by LPL and hepatic lipase. Here the distribution of lipoprotein subclasses is consistent with the kinetic model of Berneis et al. during high hepatic triglyceride availability. In this state, the production of very large, triglyceride-rich VLDL particles is increased. High concentration in triglyceride-rich VLDL and longer plasmatic half time in turn leads to augmented HL hydrolyzation resulting in small LDL particles. The elevated triglyceride levels in HDL3-4 are well explained by increased activity of cholesterol ester transfer protein in the high hepatic triglycerides model [28]. In summary, our data support the hypothesis, that BAFF/APRIL signaling might provide a link between the immune system and metabolic pathways to provide the immune system with energy [24]. Combining our findings with the existing literature we hypothesize a pathway in which the BAFF/APRIL axis is a key player to satisfy the elevated energy demand in systemic inflammation.

### MMP2 and lipoprotein interaction

MMP-2, also known as gelatinase A, is an endopeptidase that participates in extracellular matrix remodeling and processing of cytokines like TGF-β, IL-1β and CCL7. The expression, secretion and activation of MMP-2 is regulated at various levels, most prominently by membrane-type 1 (MT1-MMP) and several tissue inhibitors of metalloproteinases (TIMPs) [29]. We detected various associations between MMP-2 and various lipoprotein subclasses. Specifically, we observed positive relations between MMP-2 and ApoA1, ApoA2 an cholesterol in the largest HDL particles and inverse associations with VLDL-ApoB, -C, -PL, and -TG especially in larger LDL particles, IDL-ApoB, -C, -PL, -TG and LDL-ApoB, -C, -PL and—TG in the smallest LDL particles.

Elevated levels of MMP2 are linked to a chronic low-grade inflammation present in cardiometabolic diseases, all of which are also associated with dyslipidemia [30, 31]. These pathologies have positive relations with total triglycerides, total cholesterol, VLDL-C and LDL-C and inverse associations with HDL-C. Our findings fit well with the recently discovered heart-liver axis, which is mediated by a secreted cardiac-specific phospholipase A2 (scPLA2). The knockout or inhibition of MMP2 in mice was dose‐dependently associated with reduced cleavage of chemokine CCL7 and subsequent, up to 1000-fold increased cardiac release of scPLA2. This scPLA2 in turn increased hepatic triglycerides, VLDL-TG and decreased HDL-C in MMP2 -/- mice [32]. Hepatocytes of MMP2 -/- mice had reduced levels of liver X receptor-α and suppressed mRNA expression of fatty acid synthase, acetyl-CoA carboxylase 1 and 2, LDL receptor-related protein 1 and fatty acid translocase. Meanwhile intrahepatic levels of sterol regulatory element-binding protein 2 and mRNA expression of HMG-CoA reductase, cholesterol 7α‐hydroxylase, sterol 27-hydroxylase, ATP‐binding cassette G5/G8 and LDL receptor (LDLR) was increased. Additionally, in vitro experiments have shown that MMP-2 can proteolytically cleave and inactivate PCSK9, a protein that facilitates LDLR degradation [33]. However, while overexpression of MMP2 protected Hepa1-c1c7 cells from PCSK9 induced LDLR degradation, there is yet no in vivo evidence of MMP2 affecting systemic lipid metabolism via a PCSK9-LDLR pathway. In summary, our findings suggest that an increased de-novo lipogenesis and hepatic FFA uptake and reduced VLDL clearance and biliary cholesterol elimination in MMP2 deficiency [34].

### Inflammatory cluster 1

Among the markers in our inflammatory panel, we observed 10 to exhibit remarkably similar associations with the investigated lipoprotein sub fractions together with strongly correlated within cluster plasma concentrations. This cluster including IFN-alpha2, IFN-gamma, IL-11, IL-12p40, IL-29, IL-32, IL-35, TSLP and MMP-1 was directly associated with ApoB, -C, -PL, and -TG primarily in large LDL1, small LDL4-5 and IDL particles. Additional significant association with VLDL particles were observed with IL-12P40, IL-32, IFN-gamma, IFN-alpha2. Plasma levels of IFN-alpha2, IFN-gamma, IL-11, IL-12p40, IL-29, IL-32, TSLP and MMP-1 are implicated in various chronic inflammatory diseases like type 2 diabetes, nonalcoholic fatty liver disease, atherosclerosis [35–42]. Albeit depending on the pathophysiological context, these cytokines are considered pro-inflammatory (IL-12p40, IL-29, IFN-alpha2, IFN-gamma, TSLP), anti-inflammatory (IL-35) or both (IL-11) [43].

Previously, IFN-alpha2 was shown to increase de-novo lipogenesis and VLDL-C secretion in hepatocytes and patients following IFN-alpha2 application [44]. Similarly, IFN-gamma treatment resulted in a dose-dependent increase in serum triglycerides [45]. Patients with hypertriglyceridemia, coronary heart disease, hypercholesteremia or major depression show positive associations between IFN-gamma and total triglycerides, total cholesterol and LDL-C. While previous studies explored the relation between IL-12 and the lipid metabolism, there is conflicting evidence with some reporting positive [46, 47], negative or no associations at all [48, 49]. Similarly, the administration of Ustekinumab, a monoclonal IL-12p40/IL-23 antibody, increased VLDL/IDL/LDL particles, LDL-C, total cholesterol and total triglycerides in some but not all clinical trials [50, 51].

Reports about associations between Il-35 and lipid parameters are conflicting. While we found positive associations between the two, some authors reported IL-35 to be negatively correlated with total cholesterol and LDL-C concentrations and positively correlated with HDL-C [52]. However, others could not find any significant associations [53].

Due to the cross-sectional study design and single-time lipoprotein/biomarker measurement, we can only speculate whether our observations are based on an asymptomatic acute infection or chronic low-grade inflammation. Yet, we assessed a cohort without mayor metabolic or autoinflammatory diseases. Moreover, many diseases linked to chronic low-grade inflammation such as atherosclerosis, metabolic associated fatty liver disease (MAFLD) or type II diabetes are thought to share similar pathways in early stages. Thus, our findings may be of valuable insight into conserved immunometabolism pathways.

### Strength and limitations

The strengths of our study include measurement of a broad number of inflammatory biomarkers and the use of ^1^H-NMR for the quantification of lipoprotein subclasses. ^1^H-NMR is a fast, high throughput method that is already used in clinical lipoprotein measurements and mayor clinical trials. Thus, our results are of high validity when compared to current and future subclass assessments. An additional strength of the present study is the study population as subsample of a large population-based cohort. Most studies dealing with lipoprotein-cytokine interactions have used patient populations, selected according to a specific, underlying condition. This however might disguise principal pathophysiologic processes of as they are accompanied by later compensatory processes or voided by preceding tissue damage.

Nonetheless, our findings are also subjected to several limitations. First, NMR is cannot differentiate between lipoprotein(a) and LDL particles which may influence the results. The cross-sectional study design does not allow for the assessment of causal relations. Studies have shown that ethnicity greatly influences cytokine gene polymorphism distribution and thus expression pattern. Therefore, the findings in this predominantly white European cohort may have limited validity for individuals of non-white ethnicity [54, 55]. Additionally, several of the biomarkers we assessed are known to have different activity states, subvariants or are part of bigger hetero multimeric proteins with diverging downstream pathways. Amongst others this is especially the case for MMP2, IL-12p40 and IL-32. Therefore, the interpretation of our result should be done with cautious regard of our methodological limitations. Lastly, future studies need to assess whether the identified clusters may be used as biomarkers for CVD risk.

## Conclusions

We identified associations between NMR-based lipoprotein subclasses and a broad inflammatory profile in an apparently healthy human population sample. Our findings revealed complex and divergent relationships between inflammatory markers and lipoprotein characteristics, which are not adequately represented in standard lipid assessment. With this data, we are able to report on several lipoprotein association which have not yet been observed in a healthy cohort. In this, we are contributing to the existing literature of immunometabolism which may help us understand early pathophysiological processes in CVD, T2D or MAFLD. Further research needs to be done to elude the directionality and the underlying mechanisms of the relations we found.

## Supplementary Information

**Additional file 1: Table S1. **Concentration of inflammatory markers in the study population.

## Data Availability

The Study of Health in Pomerania (SHIP) data are publicly available for scientific and quality control purposes. Data usage can be applied for via www.community-medicine.de.

## Abbreviations

ALAT: Alanine aminotransferase
ApoA: Apolipoprotein A
ApoB: Apolipoprotein B
ASAT: Aspartate aminotransferase
ATC: Anatomical Therapeutic Chemical
BAFF: B-cell activating factor
BCMA: B-cell maturation antigen
BIA: Bioelectrical impedance analysis
BMI: Body mass index
C: Cholesterol
CCL7: C-C motif chemokine ligand 7
CVD: Cardiovascular disease
DAMP: Damage-associated molecular pattern
eGFR: Estimated glomerular filtration rate
FDR: False discovery rate
FFA: Free fatty acids
GGT: Gamma-glutamyl transferase
GN: Glomerulonephritis
gp130: Glycoprotein 130
HBA1c: Hemoglobin A1c
HDL: High-density lipoprotein
HL: Hepatic lipase
HMG-CoA Reductase: 3-hydroxy-3-methylglutaryl-coenzyme A reductase
IBM: Inflammatory biomarker
IDL: Intermediate-density lipoprotein
IFN: Interferon
IL: Interleukin
IVS: Interventricular septum
LDL: Low-density lipoprotein
LDL-R: LDL receptor
LOX-1: Lectin-like oxidized low-density lipoprotein receptor 1
LVDD: Left ventricular diastolic dysfunction
LVDS: Left ventricular diastolic function
LVMI: Left ventricular mass index
LVPW: Left ventricular posterior wall
MAFLD: Metabolic associated fatty liver disease
MMP: Matrix metalloproteinase
mRNA: Messenger RNA
MT1-MMP: Membrane-type 1 matrix metalloproteinase
NMR: Nuclear magnetic resonance
PAMP: Pathogen-associated molecular pattern
PRR: Pattern recognition receptor
PCSK9: Proprotein convertase subtilisin/kexin type 9
PL: Phospholipids
sAPRIL: Soluble a proliferation-inducing ligand
sBAFF: Soluble B-cell activating factor
sCD: Soluble cluster of differentiation
scPLA2: Secretory phospholipase A2
SHIP: Study of Health in Pomerania
T2D: Type 2 diabetes
TACI: Transmembrane activator and calcium modulator and cyclophilin ligand interactor
TC: Total cholesterol
TG: Triglycerides
TGF: Transforming growth factor
TIMPs: Tissue inhibitors of metalloproteinases
TLR: Toll-like receptor
TNF: Tumor necrosis factor
TSLP: Thymic stromal lymphopoietin
TWEAK: TNF-like weak inducer of apoptosis
VLDL: Very-low-density lipoprotein
