# Supplementary material for: Low-grade inflammation is associated with a heterogeneous lipoprotein subclass profile in an apparently healthy population sample
Source: Lipids Health Dis. 2023 Jul 11;22:100. doi: 10.1186/s12944-023-01856-6 (PMC10334607; doi:10.1186/s12944-023-01856-6)
Supplement: Supplementary file 1 — Additional file 1: Table S1. Concentration of inflammatory markers in the study population. [file 12944_2023_1856_MOESM1_ESM.pdf]

Supplement

"Low-grade inflammation is associated with a heterogeneous lipoprotein subclass profile in apparently healthy population sample"

Table S1. Concentration of inflammatory markers in the study population.

	Coefficient of variation [%]	**Concentration of inflammatory markers [pg/ml]**				
		**Median**	**Q1**	**Q3**	**Mean**	**SD**
Chitinase 3-like 1	*11.2*	8792	7208	10459	8971	2623
IFN-alpha2	*10.2*	44.7	38.5	55.3	49.2	16.1
IFN-beta	*6.2*	21.2	15.9	26.2	22.9	13.7
IFN-gamma	*9.6*	10.2	8.5	13.6	12.0	5.4
IL-10	*5.9*	24.2	22.2	26.4	24.2	3.8
IL-11	*15.8*	1.16	0.78	1.86	1.43	0.96
IL-12 (p40)	*5.5*	63.1	48.4	90.1	75.0	37.3
IL-19	*7.3*	35.3	31.7	40.9	37.6	9.4
IL-22	*8.9*	19.7	17.6	22.5	23.3	30.9
IL-26	*9.7*	101.7	96.0	107.8	101.3	15.4
IL-29 / IFN-lambda1	*9*	76.3	60.4	101.9	85.7	43.2
IL-32	*7.3*	32.1	21.6	54.1	43.4	35.5
IL-34	*7.4*	170.1	120.2	232.6	192.1	106.1
IL-35	*3.4*	124.8	101.7	163.7	140.9	56.4
IL-8	*7.7*	200.1	169.5	240.4	218.4	86.8
MMP-1	*10.3*	891.3	699.2	1094.7	931.1	374.9
MMP-2	*7.7*	4399	3575	5291	4758	1897
MMP-3	*6.6*	5812	4025	7940	6268	2909
Osteocalcin	*5*	2024	1679	2521	2146	750
Osteopontin (OPN)	*11.5*	30248	24857	37022	34733	20744
Pentraxin-3	*10.3*	360.9	251.2	486.1	416.7	267.0
TWEAK / TNFSF12	*20.9*	210.0	179.9	246.3	214.2	53.0
APRIL / TNFSF13	*20.2*	106158	89479	127615	110169	32524
BAFF / TNFSF13B	*3.7*	5586	4819	6786	5963	1947
LIGHT / TNFSF14	*9.1*	75.7	52.6	107.7	90.9	72.3
TSLP	*9.8*	62.0	52.7	82.1	71.7	32.4
gp130 / sIL-6Rbeta	*4.7*	15953	12693	19201	16972	7887
sCD163	*9.7*	49902	37794	64626	55050	27411
sCD30 / TNFRSF8	*4.9*	213.2	162.0	273.6	231.9	109.0
sTNF-R1	*4.1*	904.6	701.4	1116.7	943.1	373.6
sTNF-R2	*4.1*	1967	1447	2494	2127	974

SD =standard deviation.

positive assocation with *FDR <0.05

inverse assocation with *FDR <0.05

Table S2. Associations of lipoprotein subclasses with inflammatory parameters assessed by linear regression analyses. All analyses were adjusted for age, sex, smoking behavior and lean body mass. Orange and blue shading indicate positive and inverse associations.

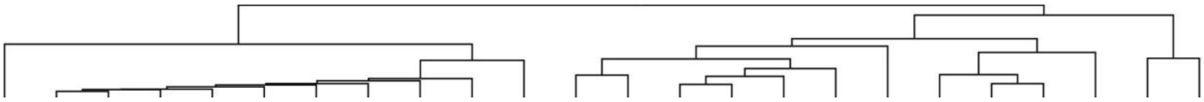

Beta estimates (stderr) for standardized outcome and exposure

	BAFF / TNFSF13B	IFN-alpha2	IFN-gamma	IL-32	IL-35	IL-12 (p40)	TSLP	MMP-1	IL-11	IL-29 / IFN-lambda1	IL-22	APRIL / TNFSF13	TWEAK / TNFSF12	LIGHT / TNFSF14	sTNF-R1	sCD163	sTNF-R2	Osteocalcin	Chitinase 3-like 1	Osteopontin (OPN)	gp130 / sIL-6Rbeta	Pentraxin-3	IL-26	MMP-2
Total Cholesterol	0.03832 (0.04705)	0.20270 (0.04635)*	0.21184 (0.04609)*	0.18757 (0.04698)*	0.20810 (0.04585)*	0.23072 (0.04579)*	0.18740 (0.04608)*	0.18533 (0.05018)*	0.21803 (0.05148)*	0.17894 (0.04683)*	0.12355 (0.04641)*	-0.02721 (0.05252)	0.06382 (0.04796)	-0.05832 (0.04972)	-0.09390 (0.04795)	-0.06306 (0.04834)	-0.14404 (0.04698)*	-0.08690 (0.04758)	-0.09426 (0.04716)	-0.06588 (0.04746)	-0.09338 (0.04719)	-0.08642 (0.04843)	-0.05285 (0.04700)	-0.11565 (0.04717)
VLDL Cholesterol	0.15934 (0.04595)*	0.11738 (0.04669)	0.12340 (0.04645)*	0.11607 (0.04841)	0.09432 (0.04635)	0.11966 (0.04638)	0.10069 (0.04631)	0.10394 (0.05112)	0.08463 (0.05339)	0.07892 (0.04723)	0.02155 (0.04637)	0.05443 (0.05202)	0.07968 (0.04749)	0.07428 (0.04998)	0.05236 (0.04772)	0.06559 (0.04791)	0.08044 (0.04694)	-0.02791 (0.04730)	0.11397 (0.04659)	0.06810 (0.04706)	0.04175 (0.04692)	-0.04122 (0.04820)	-0.12389 (0.04622)*	-0.18837 (0.04614)*
VLDL-1 Cholesterol	0.15471 (0.04664)*	0.11102 (0.04719)	0.10647 (0.04697)	0.10436 (0.04871)	0.08423 (0.04689)	0.10524 (0.04694)	0.09584 (0.04674)	0.08105 (0.05175)	0.06534 (0.05456)	0.06417 (0.04765)	0.03627 (0.04672)	0.07853 (0.05295)	0.08140 (0.04796)	0.09107 (0.05051)	0.07143 (0.04811)	0.08045 (0.04844)	0.09895 (0.04726)	-0.00529 (0.04819)	0.13415 (0.04694)*	0.05330 (0.04765)	0.03143 (0.04738)	-0.05649 (0.04901)	-0.14223 (0.04832)*	-0.19817 (0.04643)*
VLDL-2 Cholesterol	0.14088 (0.04650)*	0.11490 (0.04715)	0.12519 (0.04693)*	0.11129 (0.04881)	0.09295 (0.04706)	0.11665 (0.04708)	0.10799 (0.04669)	0.08318 (0.05237)	0.08017 (0.05501)	0.07470 (0.04763)	0.03785 (0.04672)	0.05637 (0.05259)	0.11007 (0.04777)	0.07135 (0.05058)	0.05656 (0.04866)	0.07996 (0.04869)	0.09542 (0.04754)	0.00167 (0.04786)	0.11935 (0.04696)	0.08985 (0.04768)	0.05104 (0.04744)	-0.04335 (0.04861)	-0.10617 (0.04693)	-0.18722 (0.04681)*
VLDL-3 Cholesterol	0.13199 (0.04700)*	0.11884 (0.04755)	0.12848 (0.04729)*	0.11845 (0.04939)	0.09950 (0.04742)	0.12978 (0.04741)*	0.10173 (0.04707)	0.10596 (0.05225)	0.09952 (0.05487)	0.07600 (0.04801)	0.02008 (0.04714)	0.04618 (0.05306)	0.10016 (0.04822)	0.06021 (0.05114)	0.05143 (0.04908)	0.06448 (0.04915)	0.07773 (0.04800)	-0.02941 (0.04818)	0.09065 (0.04749)	0.08392 (0.04806)	0.04680 (0.04782)	-0.01787 (0.04902)	-0.11614 (0.04722)	-0.18387 (0.04795)*
VLDL-4 Cholesterol	0.12745 (0.04694)*	0.11144 (0.04750)	0.12280 (0.04720)	0.11473 (0.04948)	0.09708 (0.04707)	0.12698 (0.04709)*	0.09966 (0.04706)	0.12344 (0.05145)	0.09912 (0.05305)	0.08709 (0.04792)	-0.00067 (0.04710)	0.03819 (0.05285)	0.06248 (0.04834)	0.03011 (0.05121)	0.01769 (0.04852)	0.04439 (0.04871)	0.03947 (0.04779)	-0.05236 (0.04803)	0.06715 (0.04755)	0.05265 (0.04784)	0.03810 (0.04765)	0.00054 (0.04898)	-0.11244 (0.04700)	-0.13828 (0.04796)*
VLDL-5 Cholesterol	0.08480 (0.05029)	0.00388 (0.05228)	0.05809 (0.05385)	0.06592 (0.05615)	0.03119 (0.05128)	0.04591 (0.05242)	0.05234 (0.05343)	0.08422 (0.05568)	0.04112 (0.05761)	-0.00484 (0.05091)	-0.04757 (0.04896)	0.03207 (0.05647)	-0.04247 (0.05153)	-0.02295 (0.05338)	0.04387 (0.05167)	0.01557 (0.05135)	0.03068 (0.05068)	-0.05671 (0.05219)	0.05928 (0.05102)	0.04431 (0.05019)	0.01395 (0.05039)	0.02875 (0.05250)	-0.03449 (0.05014)	-0.10525 (0.05001)
IDL Cholesterol	0.13441 (0.04579)*	0.18785 (0.04568)*	0.19011 (0.04550)*	0.17930 (0.04726)*	0.15283 (0.04557)*	0.19528 (0.04537)*	0.16368 (0.04548)*	0.17378 (0.04950)*	0.15690 (0.05201)*	0.14665 (0.04638)*	0.05732 (0.04595)	0.02661 (0.05166)	0.04386 (0.04719)	0.02675 (0.04918)	-0.01846 (0.04729)	0.00433 (0.04764)	-0.00989 (0.04672)	-0.09254 (0.04673)	0.03081 (0.04655)	-0.01070 (0.04682)	-0.01304 (0.04661)	-0.05652 (0.04774)	-0.12505 (0.04584)*	-0.16492 (0.04599)*
LDL Cholesterol	-0.00791 (0.04769)	0.17330 (0.04727)*	0.17854 (0.04706)*	0.15524 (0.04819)*	0.17729 (0.04679)*	0.19225 (0.04685)*	0.14286 (0.04709)*	0.15051 (0.05078)*	0.20658 (0.05232)*	0.14374 (0.04782)*	0.12654 (0.04698)*	-0.01221 (0.05325)	0.06761 (0.04857)	-0.05634 (0.05003)	-0.08851 (0.04864)	-0.08266 (0.04891)	-0.15156 (0.04754)*	-0.08218 (0.04822)	-0.16226 (0.04731)*	-0.10691 (0.04787)	-0.13483 (0.04756)*	-0.12195 (0.04891)	-0.03142 (0.04766)	-0.09352 (0.04791)
LDL-1 Cholesterol	-0.01118 (0.04750)	0.13360 (0.04739)*	0.15712 (0.04706)*	0.11279 (0.04868)	0.15182 (0.04681)*	0.14842 (0.04705)*	0.15123 (0.04683)*	0.11548 (0.05182)	0.13281 (0.05318)	0.16396 (0.04745)*	0.09029 (0.04705)	-0.00047 (0.05299)	0.04612 (0.04843)	-0.09586 (0.05033)	-0.13218 (0.04820)*	-0.03341 (0.04876)	-0.13417 (0.04747)*	0.01360 (0.04819)	-0.11872 (0.04743)	-0.11491 (0.04757)	-0.10032 (0.04757)	-0.13014 (0.04861)*	-0.03806 (0.04745)	-0.01742 (0.04794)
LDL-2 Cholesterol	-0.05750 (0.04880)	0.07143 (0.04880)	0.09314 (0.04863)	0.06440 (0.04985)	0.07596 (0.04830)	0.07223 (0.04859)	0.07539 (0.04853)	0.02711 (0.05347)	0.08252 (0.05312)	0.09820 (0.04919)	0.04292 (0.04822)	-0.00868 (0.05410)	0.04556 (0.04951)	-0.09925 (0.05124)	-0.11402 (0.04940)	-0.09175 (0.04967)	-0.15962 (0.04835)*	0.04149 (0.04918)	-0.17781 (0.04802)*	-0.13298 (0.04852)*	-0.10833 (0.04857)	-0.11143 (0.04978)	0.00364 (0.04858)	0.07136 (0.04884)
LDL-3 Cholesterol	-0.04913 (0.04862)	0.12021 (0.04849)	0.12896 (0.04827)*	0.10213 (0.04972)	0.13175 (0.04796)*	0.11938 (0.04825)	0.10121 (0.04815)	0.09719 (0.05206)	0.15365 (0.05295)*	0.11844 (0.04882)	0.10057 (0.04797)	-0.02742 (0.05410)	0.02566 (0.04922)	-0.08594 (0.05091)	-0.12430 (0.04930)	-0.12935 (0.04944)*	-0.19562 (0.04796)*	-0.04700 (0.04915)	-0.20676 (0.04770)*	-0.16219 (0.04836)*	-0.15784 (0.04819)*	-0.14694 (0.04956)*	0.00868 (0.04847)	0.00808 (0.04893)
LDL-4 Cholesterol	0.00073 (0.04792)	0.17335 (0.04750)*	0.16225 (0.04744)*	0.13728 (0.04898)*	0.16846 (0.04710)*	0.17352 (0.04726)*	0.12647 (0.04743)*	0.18082 (0.05036)*	0.22267 (0.05283)*	0.13277 (0.04817)*	0.12686 (0.04724)*	-0.05403 (0.05344)	-0.00030 (0.04892)	-0.04250 (0.05091)	-0.10751 (0.04881)	-0.12496 (0.04894)	-0.17616 (0.04756)*	-0.15953 (0.04796)*	-0.18822 (0.04730)*	-0.13076 (0.04788)*	-0.16577 (0.04754)*	-0.12819 (0.04911)*	-0.01491 (0.04790)	-0.09208 (0.04817)
LDL-5 Cholesterol	0.05317 (0.04486)	0.13187 (0.04480)*	0.12325 (0.04472)*	0.12267 (0.04618)*	0.12221 (0.04445)*	0.14923 (0.04444)*	0.08963 (0.04466)	0.13093 (0.04836)*	0.16625 (0.04971)*	0.07051 (0.04547)	0.09080 (0.04441)	0.00414 (0.05017)	0.05398 (0.04580)	0.02368 (0.04734)	0.00509 (0.04602)	-0.01810 (0.04624)	-0.03078 (0.04534)	-0.12090 (0.04520)*	-0.05331 (0.04515)	-0.00279 (0.04526)	-0.05521 (0.04518)	-0.04314 (0.04638)	-0.04448 (0.04488)	-0.14988 (0.04475)*
LDL-6 Cholesterol	0.03406 (0.04613)	0.08218 (0.04633)	0.08468 (0.04617)	0.09662 (0.04772)	0.08496 (0.04589)	0.12108 (0.04589)*	0.06387 (0.04598)	0.06472 (0.05086)	0.09054 (0.05137)	0.03280 (0.04683)	0.06798 (0.04568)	0.03758 (0.05151)	0.09362 (0.04688)	0.04200 (0.04944)	0.06780 (0.04709)	0.03682 (0.04749)	0.03937 (0.04655)	-0.03500 (0.04680)	0.02991 (0.04643)	0.05405 (0.04656)	-0.00023 (0.04649)	0.00390 (0.04771)	-0.03742 (0.04611)	-0.15495 (0.04596)*
HDL Cholesterol	-0.07641 (0.04492)	0.02743 (0.04540)	0.02226 (0.04526)	0.02050 (0.04641)	0.05390 (0.04493)	0.05210 (0.04513)	0.05838 (0.04492)	0.03591 (0.04766)	0.03817 (0.04891)	0.06294 (0.04555)	0.01305 (0.04483)	-0.13669 (0.04965)*	-0.07803 (0.04585)	-0.12388 (0.04706)	-0.12660 (0.04575)*	-0.09022 (0.04605)	-0.16564 (0.04474)*	-0.02990 (0.04571)	-0.04940 (0.04530)	-0.02253 (0.04547)	-0.00065 (0.04540)	0.10473 (0.04609)	0.08537 (0.04486)	0.13406 (0.04499)*
HDL-1 Cholesterol	-0.04931 (0.04457)	0.01888 (0.04497)	0.03327 (0.04480)	0.03689 (0.04600)	0.06242 (0.04446)	0.05561 (0.04473)	0.06324 (0.04446)	-0.00055 (0.04699)	0.00931 (0.04906)	0.06889 (0.04511)	0.00536 (0.04441)	-0.07222 (0.04952)	-0.04797 (0.04550)	-0.09321 (0.04707)	-0.09953 (0.04547)	-0.05674 (0.04574)	-0.07499 (0.04490)	0.03988 (0.04524)	-0.03020 (0.04490)	-0.00995 (0.04502)	0.00714 (0.04495)	0.09138 (0.04573)	0.05990 (0.04452)	0.14041 (0.04451)*
HDL-2 Cholesterol	-0.03586 (0.04420)	0.02313 (0.04456)	0.03014 (0.04440)	0.03038 (0.04587)	0.05644 (0.04408)	0.05387 (0.04428)	0.05596 (0.04408)	0.04792 (0.04598)	0.02881 (0.04798)	0.05456 (0.04475)	-0.01744 (0.04398)	-0.14434 (0.04869)*	-0.09287 (0.04491)	-0.10336 (0.04656)	-0.12514 (0.04489)*	-0.06954 (0.04536)	-0.12584 (0.04420)*	-0.05623 (0.04479)	-0.02577 (0.04450)	-0.00994 (0.04464)	0.03376 (0.04452)	0.13416 (0.04506)*	0.05374 (0.04414)	0.13466 (0.04414)*
HDL-3 Cholesterol	-0.02767 (0.04465)	0.03629 (0.04498)	0.02661 (0.04485)	0.02631 (0.04646)	0.05428 (0.04452)	0.05401 (0.04472)	0.06484 (0.04449)	0.08072 (0.04725)	0.06038 (0.04885)	0.04998 (0.04518)	0.01185 (0.04437)	-0.16855 (0.04903)*	-0.11638 (0.04523)	-0.12302 (0.04731)	-0.11747 (0.04540)	-0.06816 (0.04579)	-0.17635 (0.04422)*	-0.12845 (0.04487)*	-0.01961 (0.04495)	-0.01848 (0.04516)	0.01995 (0.04498)	0.11681 (0.04567)	0.06211 (0.04455)	0.09295 (0.04481)
HDL-4 Cholesterol	-0.08888 (0.04946)	0.00775 (0.05004)	-0.03296 (0.04985)	-0.03834 (0.05166)	-0.01240 (0.04958)	-0.00240 (0.04980)	-0.01208 (0.04959)	0.02589 (0.05373)	0.04571 (0.05580)	-0.00513 (0.05040)	0.05615 (0.04934)	-0.07326 (0.05532)	-0.00884 (0.05069)	-0.06092 (0.05200)	-0.02570 (0.05086)	-0.07154 (0.05095)	-0.18012 (0.04931)*	-0.05926 (0.05030)	-0.05365 (0.04991)	-0.03939 (0.05004)	-0.05542 (0.04994)	-0.03868 (0.05127)	0.06762 (0.04953)	-0.03534 (0.05006)
Total Triglycerides	0.16234 (0.04627)*	0.12052 (0.04696)	0.11800 (0.04682)	0.11650 (0.04829)	0.09699 (0.04666)	0.12718 (0.04668)*	0.10680 (0.04661)	0.10192 (0.05149)	0.07886 (0.05430)	0.06882 (0.04754)	0.03767 (0.04669)	0.04909 (0.05241)	0.03898 (0.04792)	0.07283 (0.05002)	0.06196 (0.04797)	0.05762 (0.04828)	0.08531 (0.04723)	-0.03523 (0.04764)	0.13774 (0.04678)*	0.05462 (0.04744)	0.03783 (0.04728)	-0.03753 (0.04852)	-0.11161 (0.04664)	-0.18725 (0.04650)*
VLDL Triglycerides	0.13688 (0.04615)*	0.10658 (0.04671)	0.10141 (0.04658)	0.10562 (0.04791)	0.08655 (0.04638)	0.11525 (0.04643)	0.08353 (0.04640)	0.09126 (0.05099)	0.07134 (0.05387)	0.04833 (0.04728)	0.03189 (0.04636)	0.05903 (0.05201)	0.05707 (0.04754)	0.07906 (0.04974)	0.07484 (0.04759)	0.05348 (0.04795)	0.08882 (0.04688)	-0.02421 (0.04732)	0.12672 (0.04652)*	0.04910 (0.04709)	0.02923 (0.04696)	-0.04788 (0.04816)	-0.11912 (0.04626)	-0.20059 (0.04602)*
IDL Triglycerides	0.15699 (0.04735)*	0.10743 (0.04833)	0.10425 (0.04810)	0.11542 (0.04953)	0.08759 (0.04789)	0.11088 (0.04806)	0.09811 (0.04787)	0.08137 (0.05350)	0.06036 (0.05622)	0.05448 (0.04877)	0.03591 (0.04767)	0.05014 (0.05357)	0.02691 (0.04950)	0.06984 (0.05138)	0.05929 (0.04943)	0.05450 (0.04943)	0.08209 (0.04849)	-0.02569 (0.04881)	0.15559 (0.04771)*	0.04809 (0.04859)	0.04174 (0.04851)	-0.05406 (0.04962)	-0.10854 (0.04766)	-0.18682 (0.04774)*
LDL Triglycerides	0.14771 (0.04559)*	0.16822 (0.04577)*	0.18127 (0.04549)*	0.15283 (0.04733)*	0.15456 (0.04546)*	0.19509 (0.04528)*	0.18521 (0.04518)*	0.15104 (0.04948)*	0.14666 (0.05182)*	0.13448 (0.04634)*	0.08679 (0.04575)	-0.02401 (0.05155)	-0.02661 (0.04713)	0.00867 (0.04886)	-0.01844 (0.04723)	0.02218 (0.04751)	0.03112 (0.04660)	-0.13254 (0.04639)*	0.02181 (0.04647)	0.03352 (0.04671)	0.01509 (0.04651)	0.04380 (0.04767)	-0.02749 (0.04616)	-0.09317 (0.04640)
LDL-1 Triglycerides	0.13917 (0.04607)*	0.13260 (0.04647)*	0.14393 (0.04624)*	0.10568 (0.04813)	0.11793 (0.04614)	0.15070 (0.04611)*	0.16915 (0.04575)*	0.12061 (0.05036)	0.09382 (0.05298)	0.12962 (0.04678)*	0.06380 (0.04622)	-0.02116 (0.05202)	-0.06937 (0.04743)	-0.01980 (0.04957)	-0.02819 (0.04764)	0.02811 (0.04795)	0.01726 (0.04702)	-0.08733 (0.04707)	0.04468 (0.04684)	0.01251 (0.04713)	0.02040 (0.04691)	0.03080 (0.04813)	-0.01344 (0.04657)	-0.04108 (0.04699)
LDL-2 Triglycerides	0.05716 (0.04652)	0.13393 (0.04648)*	0.16157 (0.04611)*	0.11826 (0.04789)	0.14614 (0.04596)*	0.16555 (0.04600)*	0.17029 (0.04576)*	0.11106 (0.04988)	0.12465 (0.05176)	0.14369 (0.04668)*	0.07314 (0.04621)	-0.07027 (0.05193)	-0.07481 (0.04743)	-0.06586 (0.04939)	-0.10974 (0.04743)	-0.03981 (0.04794)	-0.05367 (0.04697)	-0.08385 (0.04710)	-0.10147 (0.04663)	-0.06069 (0.04704)	-0.04301 (0.04689)	-0.00639 (0.04817)	0.01240 (0.04659)	0.01782 (0.04706)
LDL-3 Triglycerides	0.11141 (0.04560)	0.15056 (0.04567)*	0.17533 (0.04530)*	0.14568 (0.04670)*	0.16058 (0.04516)*	0.17969 (0.04518)*	0.16915 (0.04513)*	0.16493 (0.04886)*	0.16423 (0.05110)*	0.13741 (0.04602)*	0.08190 (0.04552)	-0.02451 (0.05128)	-0.06457 (0.04678)	-0.05162 (0.04859)	-0.07458 (0.04690)	-0.03541 (0.04728)	-0.05023 (0.04630)	-0.12084 (0.04621)*	-0.05218 (0.04616)	-0.04257 (0.04642)	-0.04998 (0.04619)	-0.00185 (0.04747)	-0.00070 (0.04592)	-0.01861 (0.04636)
LDL-4 Triglycerides	0.12615 (0.04596)*	0.17961 (0.04587)*	0.18905 (0.04562)*	0.15413 (0.04757)*	0.16293 (0.04560)*	0.19195 (0.04552)*	0.17737 (0.04547)*	0.17961 (0.04958)*	0.18258 (0.05184)*	0.13981 (0.04655)*	0.09183 (0.04593)	-0.04508 (0.05169)	-0.03631 (0.04732)	0.00392 (0.04927)	-0.05828 (0.04743)	-0.02071 (0.04769)	-0.00770 (0.04683)	-0.17620 (0.04624)*	-0.04524 (0.04664)	-0.00478 (0.04685)	-0.03285 (0.04669)	0.01249 (0.04789)	-0.02209 (0.04637)	-0.08627 (0.04663)
LDL-5 Triglycerides	0.12985 (0.04519)*	0.13704 (0.04549)*	0.14417 (0.04528)*	0.13318 (0.04702)*	0.12243 (0.04517)*	0.15494 (0.04512)*	0.15010 (0.04512)*	0.12175 (0.04993)	0.13205 (0.05089)	0.08739 (0.04613)	0.07546 (0.04526)	0.01557 (0.05093)	0.02899 (0.04658)	0.04852 (0.04867)	0.02215 (0.04672)	0.03998 (0.04690)	0.06710 (0.04596)	-0.10828 (0.04600)	0.04238 (0.04590)	0.05904 (0.04604)	0.01695 (0.04597)	0.01454 (0.04714)	-0.05007 (0.04557)	-0.14295 (0.04552)*
LDL-6 Triglycerides	0.11723 (0.04596)	0.10022 (0.04642)	0.10875 (0.04622)	0.11414 (0.04774)	0.08767 (0.04606)	0.13083 (0.04600)*	0.09581 (0.04602)	0.08417 (0.05104)	0.07797 (0.05265)	0.04935 (0.04696)	0.05073 (0.04596)	0.01955 (0.05174)	0.02795 (0.04728)	0.07069 (0.04920)	0.07895 (0.04721)	0.05199 (0.04761)	0.05282 (0.04658)	-0.06574 (0.04690)	0.10143 (0.04636)	0.08263 (0.04669)	0.04239 (0.04662)	0.03818 (0.04785)	-0.02835 (0.04630)	-0.16364 (0.04606)*
HDL Triglycerides	0.11838 (0.04777)	0.08111 (0.04834)	0.08741 (0.04815)	0.07497 (0.05035)	0.09083 (0.04785)	0.10886 (0.04797)	0.11629 (0.04772)	0.10290 (0.05084)	0.05077 (0.05423)	0.07281 (0.04869)	0.01211 (0.04789)	-0.04402 (0.05372)	-0.07675 (0.04899)	-0.01025 (0.05145)	-0.03723 (0.04928)	0.05068 (0.04948)	0.06760 (0.04848)	-0.03884 (0.04881)	0.12422 (0.04805)	0.10125 (0.04846)	0.09600 (0.04824)	0.12956 (0.04933)*	-0.01122 (0.04812)	0.00243 (0.04859)
HDL-1 Triglycerides	0.06383 (0.04689)	0.06802 (0.04745)	0.08583 (0.04748)	0.08006 (0.04980)	0.09183 (0.04690)	0.10945 (0.04737)	0.11220 (0.04674)	0.06675 (0.04875)	0.03520 (0.05292)	0.07952 (0.04745)	0.00318 (0.04664)	-0.04888 (0.05231)	-0.07798 (0.04805)	-0.04140 (0.04971)	-0.07370 (0.04798)	0.01463 (0.04827)	0.04046 (0.04742)	0.00429 (0.04771)	0.07379 (0.04719)	0.07188 (0.04730)	0.07444 (0.04711)	0.14193 (0.04793)*	0.02301 (0.04685)	0.07854 (0.04716)
HDL-2 Triglycerides	0.10859 (0.04763)	0.06491 (0.04820)	0.07784 (0.04800)	0.07080 (0.05012)	0.07890 (0.04771)	0.10194 (0.04781)	0.09986 (0.04762)	0.09980 (0.05039)	0.04118 (0.05360)	0.05993 (0.04854)	-0.01235 (0.04770)	-0.06789 (0.05343)	-0.09439 (0.04871)	-0.01540 (0.05101)	-0.04486 (0.04907)	0.04121 (0.04928)	0.06347 (0.04826)	-0.05993 (0.04855)	0.10123 (0.04799)	0.09518 (0.04828)	0.10469 (0.04800)	0.15990 (0.04890)*	-0.00988 (0.04793)	0.03670 (0.04837)
HDL-3 Triglycerides	0.14017 (0.04794)*	0.07927 (0.04866)	0.08105 (0.04849)	0.07386 (0.05054)	0.07625 (0.04822)	0.10350 (0.04831)	0.10730 (0.04808)	0.11473 (0.05216)	0.06115 (0.05457)	0.05052 (0.04906)	0.00156 (0.04819)	-0.05693 (0.05402)	-0.08189 (0.04928)	-0.00408 (0.05178)	-0.01261 (0.04963)	0.06534 (0.04969)	0.07072 (0.04878)	-0.09189 (0.04894)	0.13409 (0.04829)*	0.10637 (0.04878)	0.10981 (0.04848)	0.13196 (0.04963)*	-0.02997 (0.04841)	-0.02374 (0.04890)
HDL-4 Triglycerides	0.14574 (0.04710)*	0.07782 (0.04787)	0.06483 (0.04777)	0.05068 (0.04977)	0.05791 (0.04750)	0.07186 (0.04766)	0.08004 (0.04743)	0.09185 (0.05202)	0.06335 (0.05430)	0.03586 (0.04833)	0.03010 (0.04740)	0.01671 (0.05318)	0.00266 (0.04866)	0.04293 (0.05079)	0.04182 (0.04878)	0.08648 (0.04883)	0.07667 (0.04796)	-0.05254 (0.04829)	0.13591 (0.04749)*	0.09210 (0.04796)	0.06739 (0.04788)	0.01288 (0.04922)	-0.05347 (0.04758)	-0.13290 (0.04766)*
Phospholipids																								
VLDL Phospholipids	0.14198 (0.04573)*	0.10652 (0.04633)	0.10741 (0.04617)	0.10956 (0.04771)	0.09326 (0.04597)	0.11673 (0.04604)	0.08228 (0.04603)	0.10008 (0.05033)	0.08499 (0.05277)	0.05383 (0.04689)	0.02007 (0.04601)	0.06111 (0.05158)	0.06417 (0.04713)	0.07659 (0.04961)	0.06414 (0.04727)	0.05327 (0.04755)	0.08541 (0.04652)	-0.02844 (0.04693)	0.10531 (0.04628)	0.05010 (0.04672)	0.02265 (0.04659)	-0.05322 (0.04775)	-0.12701 (0.04583)*	-0.20184 (0.04562)*
IDL Phospholipids	0.12893 (0.04665)*	0.17367 (0.04665)*	0.17602 (0.04647)*	0.16673 (0.04779)*	0.15729 (0.04636)*	0.18664 (0.04629)*	0.15820 (0.04636)*	0.14889 (0.05071)*	0.14346 (0.05340)*	0.12773 (0.04733)*	0.07089 (0.04670)	0.02797 (0.05257)	0.07342 (0.04793)	0.03350 (0.05027)	-0.00052 (0.04816)	0.02271 (0.04848)	0.01303 (0.04753)	-0.05545 (0.04770)	0.06504 (0.04729)	0.01143 (0.04764)	0.00136 (0.04743)	-0.07587 (0.04853)	-0.12240 (0.04668)*	-0.19324 (0.04654)*
LDL Phospholipids	0.00745 (0.04746)	0.17866 (0.04698)*	0.18779 (0.04673)*	0.16134 (0.04780)*	0.18503 (0.04648)*	0.19990 (0.04653)*	0.15906 (0.04674)*	0.16001 (0.05048)*	0.21384 (0.05195)*	0.14972 (0.04752)*	0.13211 (0.04672)*	-0.02193 (0.05298)	0.04955 (0.04838)	-0.06123 (0.04970)	-0.09349 (0.04838)	-0.07722 (0.04861)	-0.14773 (0.04733)*	-0.07669 (0.04790)	-0.15533 (0.04713)*	-0.09960 (0.04770)	-0.12897 (0.04736)*	-0.11131 (0.04873)	-0.02533 (0.04743)	-0.09258 (0.04768)
HDL Phospholipids	-0.01879 (0.04346)	0.03501 (0.04376)	0.04078 (0.04361)	0.03856 (0.04503)	0.06161 (0.04329)	0.05884 (0.04349)	0.06730 (0.04321)	0.06298 (0.04500)	0.04909 (0.04701)	0.06106 (0.04390)	0.01231 (0.04319)	-0.13395 (0.04789)*	-0.11781 (0.04398)*	-0.12366 (0.04609)*	-0.12378 (0.04411)*	-0.05749 (0.04460)	-0.11935 (0.04347)*	-0.06252 (0.04399)	0.00659 (0.04375)	0.01942 (0.04388)	0.03410 (0.04374)	0.13178 (0.04426)*	0.07172 (0.04331)	0.12430 (0.04343)*
Total Apo-A1	-0.04365 (0.04541)	0.05445 (0.04573)	0.04058 (0.04561)	0.03639 (0.04694)	0.06813 (0.04526)	0.07582 (0.04543)	0.08864 (0.04518)	0.06988 (0.04765)	0.07104 (0.04926)	0.07473 (0.04586)	0.03316 (0.04517)	-0.15085 (0.05005)*	-0.11059 (0.04607)	-0.12968 (0.04858)*	-0.13430 (0.04609)*	-0.08314 (0.04654)	-0.15577 (0.04522)*	-0.06523 (0.04601)	-0.02347 (0.04574)	0.01710 (0.04594)	0.01365 (0.04578)	0.13870 (0.04633)*	0.08471 (0.04525)	0.12376 (0.04546)*
HDL-1 Apo-A1	-0.03744 (0.04488)	0.02363 (0.04524)	0.03838 (0.04507)	0.03770 (0.04645)	0.05361 (0.04477)	0.04952 (0.04497)	0.06660 (0.04473)	0.00843 (0.04703)	0.01335 (0.04952)	0.06680 (0.04541)	-0.00734 (0.04468)	-0.06902 (0.04991)	-0.05790 (0.04576)	-0.09635 (0.04722)	-0.11210 (0.04568)	-0.04999 (0.04610)	-0.05335 (0.04526)	0.04120 (0.04552)	-0.01575 (0.04520)	0.01963 (0.04532)	0.02633 (0.04521)	0.12466 (0.04588)*	0.06105 (0.04480)	0.16969 (0.04455)*
HDL-2 Apo-A1	0.00998 (0.04394)	0.03630 (0.04424)	0.03762 (0.04409)	0.04010 (0.04573)	0.05637 (0.04378)	0.05145 (0.04396)	0.08611 (0.04366)	0.06311 (0.04523)	0.04703 (0.04737)	0.05020 (0.04442)	0.01023 (0.04366)	-0.14568 (0.04839)*	-0.15002 (0.04422)*	-0.10741 (0.04675)	-0.11050 (0.04468)	-0.04999 (0.04517)	-0.08923 (0.04413)	-0.08439 (0.04438)	0.02559 (0.04420)	0.03674 (0.04437)	0.05344 (0.04417)	0.15894 (0.04459)*	0.08242 (0.04373)	0.13113 (0.04388)*
HDL-3 Apo-A1	0.00619 (0.04499)	0.04205 (0.04528)	0.03145 (0.04516)	0.03144 (0.04698)	0.05422 (0.04484)	0.05326 (0.04501)	0.07410 (0.04477)	0.09071 (0.04722)	0.06722 (0.04912)	0.04542 (0.04550)	0.01644 (0.04469)	-0.16946 (0.04940)*	-0.15547 (0.04526)*	-0.11109 (0.04788)	-0.10743 (0.04579)	-0.06830 (0.04619)	-0.14345 (0.04484)*	-0.13992 (0.04510)*	0.00709 (0.04528)	0.01175 (0.04550)	0.02838 (0.04528)	0.13584 (0.04589)*	0.06143 (0.04487)	0.08092 (0.04522)
HDL-4 Apo-A1	-0.04948 (0.04955)	0.04540 (0.04994)	-0.00211 (0.04983)	-0.01142 (0.05182)	0.01921 (0.04953)	0.03207 (0.04973)	0.02767 (0.04952)	0.06454 (0.05349)	0.08336 (0.05559)	0.02382 (0.05032)	0.08421 (0.04919)	-0.08581 (0.05523)	-0.03457 (0.05062)	-0.05583 (0.05178)	-0.03363 (0.05078)	-0.06098 (0.05096)	-0.16292 (0.04941)*	-0.08815 (0.05015)	-0.03581 (0.04990)	-0.00464 (0.04999)	-0.04980 (0.04990)	-0.00804 (0.05127)	0.06279 (0.04950)	-0.05581 (0.04999)
Total Apo-A2	-0.02492 (0.04873)	0.10217 (0.04885)	0.08062 (0.04880)	0.07931 (0.05059)	0.08924 (0.04847)	0.11439 (0.04855)	0.11745 (0.04832)	0.13918 (0.05214)*	0.11295 (0.05367)	0.06590 (0.04923)	0.04089 (0.04843)	-0.16612 (0.05372)*	-0.07868 (0.04960)	-0.11255 (0.05180)	-0.10805 (0.04961)	-0.07361 (0.05006)	-0.17203 (0.04845)*	-0.14181 (0.04895)*	0.01444 (0.04906)	0.00788 (0.04932)	0.04115 (0.04905)	0.10429 (0.05004)	0.03739 (0.04870)	0.01676 (0.04917)
HDL-1 Apo-A2	0.00799 (0.04469)	0.06541 (0.04491)	0.08637 (0.04467)	0.08834 (0.04625)	0.09059 (0.04439)	0.09422 (0.04456)	0.11307 (0.04427)	0.06723 (0.04582)	0.05742 (0.04818)	0.08047 (0.04508)	-0.00144 (0.04444)	-0.08917 (0.04957)	-0.08322 (0.04542)	-0.10031 (0.04724)	-0.11496 (0.04543)	-0.05412 (0.04592)	-0.05280 (0.04503)	-0.09669 (0.04533)	0.01719 (0.04497)	0.03220 (0.04512)	0.04705 (0.04494)	0.13834 (0.04554)*	0.04692 (0.04461)	0.13399 (0.04462)*
HDL-2 Apo-A2	0.01893 (0.04475)	0.10271 (0.04481)	0.11361 (0.04459)	0.12262 (0.04633)*	0.11508 (0.04432)	0.13473 (0.04437)*	0.13504 (0.04417)*	0.12678 (0.04617)*	0.10855 (0.04763)	0.08543 (0.04513)	-0.00032 (0.04449)	-0.14634 (0.04936)*	-0.12854 (0.04523)*	-0.10782 (0.04761)	-0.13609 (0.04535)*	-0.07893 (0.04590)	-0.09988 (0.04490)	-0.12389 (0.04499)*	0.00542 (0.04505)	0.00155 (0.04527)	0.03955 (0.04503)	0.13161 (0.04571)*	0.00444 (0.04475)	0.07706 (0.04502)
HDL-3 Apo-A2	0.04187 (0.04716)	0.11326 (0.04723)	0.10772 (0.04711)	0.10896 (0.04905)	0.11319 (0.04679)	0.13214 (0.04688)*	0.13945 (0.04662)*	0.15568 (0.05016)*	0.12035 (0.05183)	0.08417 (0.04760)	0.01398 (0.04689)	-0.16842 (0.05199)*	-0.10664 (0.04789)	-0.09908 (0.05023)	-0.11370 (0.04803)	-0.06124 (0.04849)	-0.13105 (0.04720)*	-0.15922 (0.04705)*	0.03083 (0.04749)	-0.00162 (0.04779)	0.05287 (0.04747)	0.10913 (0.04844)	-0.00635 (0.04719)	0.02035 (0.04763)
HDL-4 Apo-A2	-0.05510 (0.04872)	0.04589 (0.04912)	0.00578 (0.04902)	0.00145 (0.05081)	0.00845 (0.04873)	0.02332 (0.04892)	0.02027 (0.04872)	0.06560 (0.05285)	0.06539 (0.05542)	0.00180 (0.04953)	0.06183 (0.04846)	-0.04939 (0.05444)	0.01700 (0.04981)	-0.03937 (0.05076)	-0.00806 (0.04996)	-0.04176 (0.05020)	-0.13860 (0.04877)*	-0.06938 (0.04950)	-0.00181 (0.04912)	-0.01865 (0.04923)	-0.02468 (0.04913)	-0.04721 (0.05038)	0.02067 (0.04878)	-0.09400 (0.04901)
Total Apo-B	0.06305 (0.04556)	0.18198 (0.04510)*	0.19085 (0.04486)*	0.16737 (0.04600)*	0.17780 (0.04472)*	0.21064 (0.04456)*	0.15758 (0.04491)*	0.16920 (0.04854)*	0.19852 (0.05004)*	0.13568 (0.04580)*	0.11794 (0.04499)*	0.01179 (0.05099)	0.05864 (0.04652)	-0.01623 (0.04811)	-0.03344 (0.04668)	-0.02378 (0.04700)	-0.06372 (0.04599)	-0.09830 (0.04607)	-0.07411 (0.04581)	-0.03661 (0.04608)	-0.08041 (0.04581)	-0.07267 (0.04706)	-0.05494 (0.04557)	-0.14758 (0.04551)*
VLDL Apo-B	0.15819 (0.04552)*	0.11232 (0.04627)	0.11735 (0.04605)	0.10725 (0.04775)	0.09700 (0.04590)	0.12431 (0.04591)*	0.09081 (0.04592)	0.11068 (0.05041)	0.08645 (0.05263)	0.06772 (0.04682)	0.01574 (0.04594)	0.04797 (0.05154)	0.05347 (0.04714)	0.07045 (0.04955)	0.06068 (0.04725)	0.05346 (0.04749)	0.08662 (0.04647)	-0.04745 (0.04681)	0.09327 (0.04627)	0.05889 (0.04663)	0.03422 (0.04650)	-0.02932 (0.04776)	-0.11256 (0.04586)	-0.18351 (0.04574)*
IDL Apo-B	0.13310 (0.04586)*	0.18470 (0.04577)*	0.18827 (0.04558)*	0.17397 (0.04743)*	0.15592 (0.04561)*	0.20164 (0.04536)*	0.17189 (0.04547)*	0.18251 (0.04923)*	0.16506 (0.05170)*	0.14514 (0.04644)*	0.05838 (0.04602)	-0.01309 (0.05173)	-0.00846 (0.04730)	0.00889 (0.04908)	-0.03633 (0.04733)	-0.00558 (0.04767)	-0.01453 (0.04677)	-0.13244 (0.04655)*	0.00476 (0.04664)	-0.00962 (0.04689)	-0.01026 (0.04667)	-0.01974 (0.04787)	-0.09488 (0.04608)	-0.13349 (0.04631)*
LDL Apo-B	0.03027 (0.04665)	0.18033 (0.04615)*	0.19241 (0.04588)*	0.16794 (0.04763)*	0.18218 (0.04570)*	0.20851 (0.04562)*	0.15907 (0.04592)*	0.16177 (0.04970)*	0.20669 (0.05113)*	0.13739 (0.04681)*	0.12980 (0.04592)*	-0.00181 (0.05212)	0.05792 (0.04755)	-0.03579 (0.04912)	-0.05561 (0.04769)	-0.04645 (0.04799)	-0.09416 (0.04687)	-0.09965 (0.04709)	-0.11286 (0.04663)	-0.06260 (0.04705)	-0.10311 (0.04672)	-0.08824 (0.04803)	-0.03548 (0.04662)	-0.12226 (0.04672)*
LDL-1 Apo-B	0.00502 (0.04688)	0.13655 (0.04674)*	0.16405 (0.04636)*	0.11750 (0.04802)	0.15343 (0.04617)*	0.16142 (0.04617)*	0.16404 (0.04608)*	0.12727 (0.05088)	0.13168 (0.05253)	0.16242 (0.04681)*	0.08638 (0.04644)	-0.01376 (0.05230)	0.00763 (0.04762)	-0.09789 (0.04960)	-0.12472 (0.04760)*	-0.02655 (0.04815)	-0.11107 (0.04699)	-0.01237 (0.04756)	-0.10209 (0.04690)	-0.09374 (0.04707)	-0.08249 (0.04703)	-0.08412 (0.04823)	-0.02535 (0.04685)	-0.00789 (0.04732)
LDL-2 Apo-B	-0.06472 (0.04856)	0.07730 (0.04889)	0.09903 (0.04863)	0.07051 (0.05002)	0.08238 (0.04842)	0.08109 (0.04863)	0.07784 (0.04844)	0.03823 (0.05350)	0.09454 (0.05329)	0.09194 (0.04911)	0.04812 (0.04836)	-0.01234 (0.05428)	0.03239 (0.04965)	-0.10680 (0.05130)	-0.11633 (0.04953)	-0.08396 (0.04986)	-0.14737 (0.04857)*	0.02861 (0.04936)	-0.17695 (0.04817)*	-0.11934 (0.04878)	-0.10073 (0.04875)	-0.08996 (0.05004)	0.01407 (0.04865)	0.07889 (0.04894)
LDL-3 Apo-B	-0.04554 (0.04843)	0.13584 (0.04837)*	0.14864 (0.04812)*	0.11712 (0.04967)	0.14508 (0.04786)*	0.14007 (0.04811)*	0.12048 (0.04804)	0.11494 (0.05195)	0.16703 (0.05291)*	0.13012 (0.04872)*	0.10892 (0.04792)	-0.03255 (0.05409)	0.01957 (0.04948)	-0.09046 (0.05085)	-0.12828 (0.04926)	-0.12507 (0.04946)	-0.18466 (0.04806)*	-0.06344 (0.04909)	-0.21031 (0.04764)*	-0.15492 (0.04843)*	-0.15765 (0.04818)*	-0.13310 (0.04963)*	0.00402 (0.04847)	0.00151 (0.04900)
LDL-4 Apo-B	0.02260 (0.04741)	0.18932 (0.04684)*	0.18141 (0.04676)*	0.15438 (0.04834)*	0.17823 (0.04650)*	0.19040 (0.04659)*	0.14698 (0.04678)*	0.19760 (0.04971)*	0.23420 (0.05214)*	0.14162 (0.04759)*	0.13130 (0.04672)*	-0.04903 (0.05289)	-0.00738 (0.04846)	-0.03983 (0.04946)	-0.10431 (0.04831)	-0.11278 (0.04850)	-0.15431 (0.04724)*	-0.17571 (0.04731)*	-0.17116 (0.04695)*	-0.11419 (0.04748)	-0.15341 (0.04714)*	-0.11392 (0.04867)	-0.02476 (0.04740)	-0.10147 (0.04762)
LDL-5 Apo-B	0.06658 (0.04470)	0.13622 (0.04465)*	0.13112 (0.04454)*	0.12886 (0.04602)*	0.12331 (0.04433)*	0.15538 (0.04428)*	0.09953 (0.04449)	0.13514 (0.04829)*	0.16229 (0.04946)*	0.07291 (0.04535)	0.08748 (0.04432)	0.00891 (0.05003)	0.05146 (0.04569)	0.02707 (0.04728)	0.01076 (0.04588)	-0.00366 (0.04613)	-0.01024 (0.04525)	-0.12126 (0.04508)*	-0.03225 (0.04509)	0.01137 (0.04518)	-0.03898 (0.04510)	-0.02945 (0.04628)	-0.05035 (0.04475)	-0.15352 (0.04461)*
LDL-6 Apo-B	0.05347 (0.04610)	0.08718 (0.04633)	0.09065 (0.04616)	0.09984 (0.04777)	0.08370 (0.04592)	0.12227 (0.04590)*	0.07137 (0.04598)	0.06809 (0.05101)	0.08759 (0.05158)	0.03552 (0.04684)	0.06768 (0.04572)	0.04434 (0.05152)	0.08756 (0.04694)	0.04528 (0.04943)	0.07342 (0.04709)	0.04846 (0.04749)	0.05432 (0.04654)	-0.03618 (0.04682)	0.05592 (0.04644)	0.06952 (0.04654)	0.01423 (0.04650)	0.01471 (0.04772)	-0.03959 (0.04613)	-0.15735 (0.04596)*

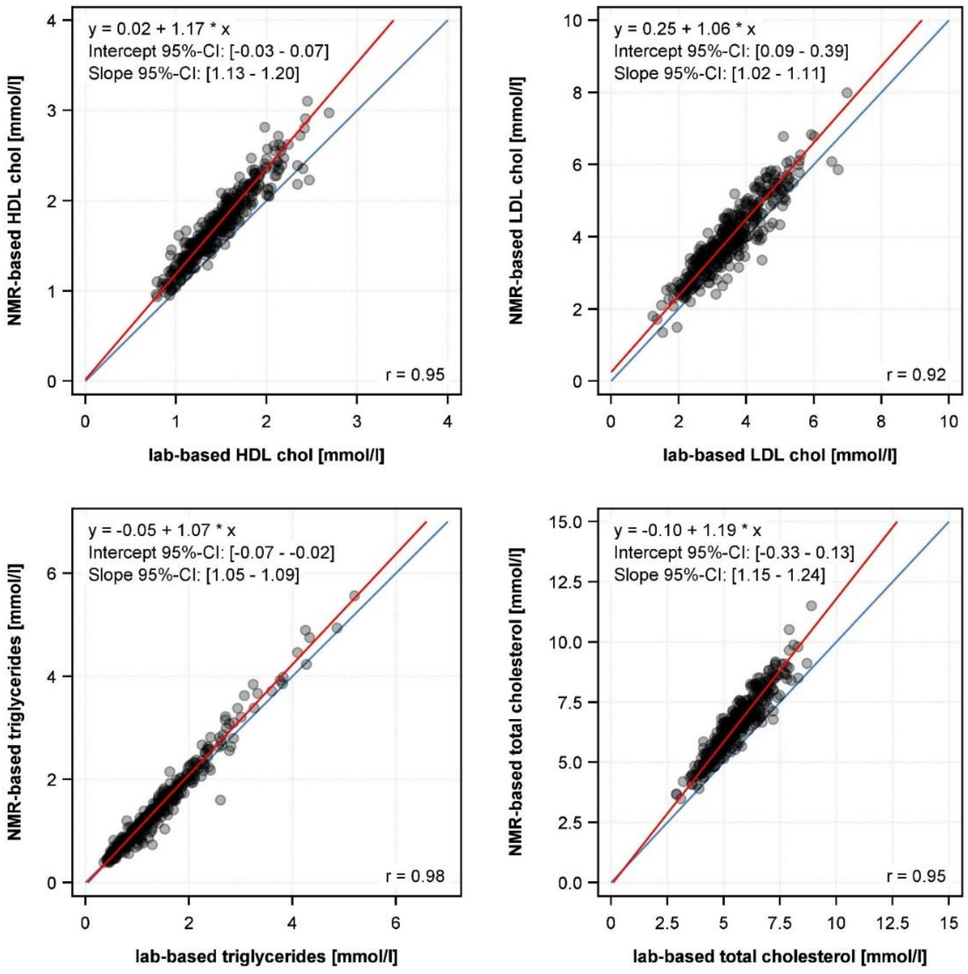

Figure S1. Comparison of the lipid levels measured by NMR spectroscopy and standard laboratory measurements. Passing-Bablok regression plots are displayed: the solid red line represents the regression line, the blue line represents the diagonal. Slope and incept are given with the 95% confidence interval (CI). Pearson correlation coefficients are given (bottom right).

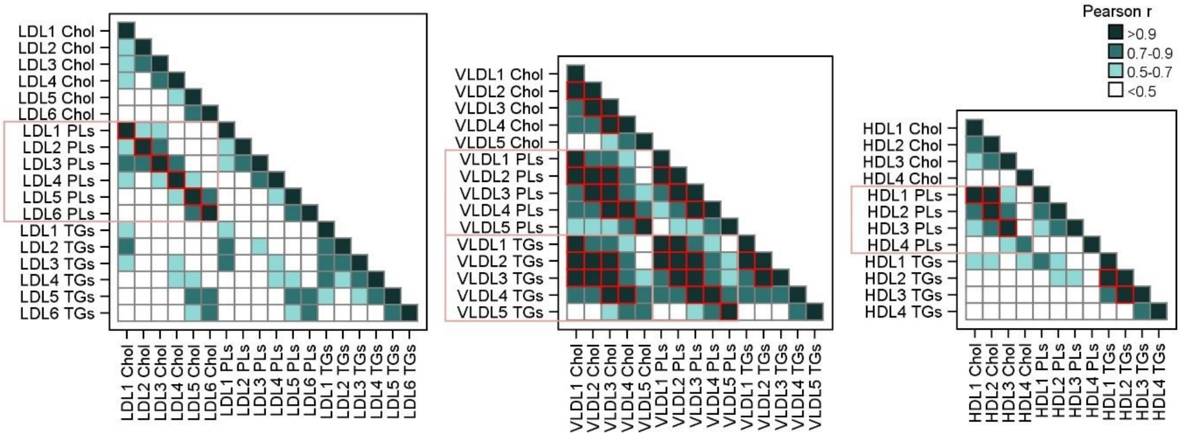

Figure S2. Correlation matrices showing within-particle Pearson correlations of cholesterol, phospholipid and triglyceride content in LDL subclasses (left), VLDL subclasses (middle) and HDL subclasses (right).

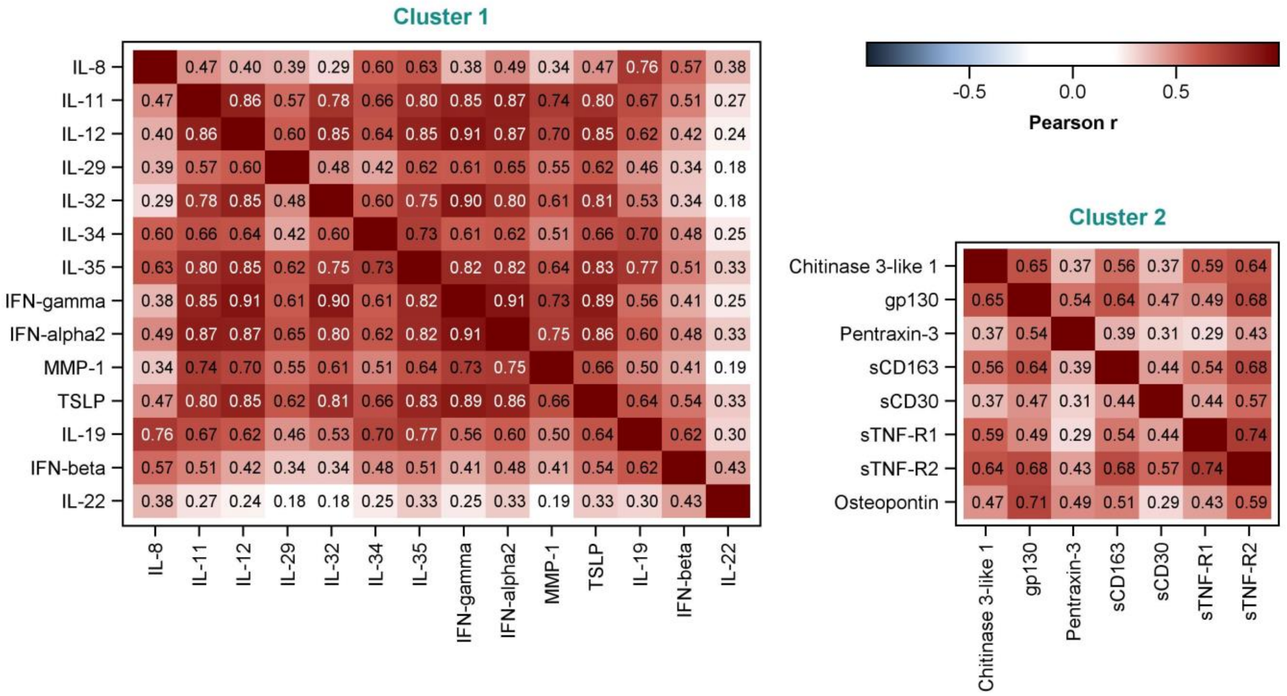

Figure S3. Color-coded Pearson correlation coefficients for the identified clusters of inflammatory markers. For all displayed correlations the p-values was < 0.01.

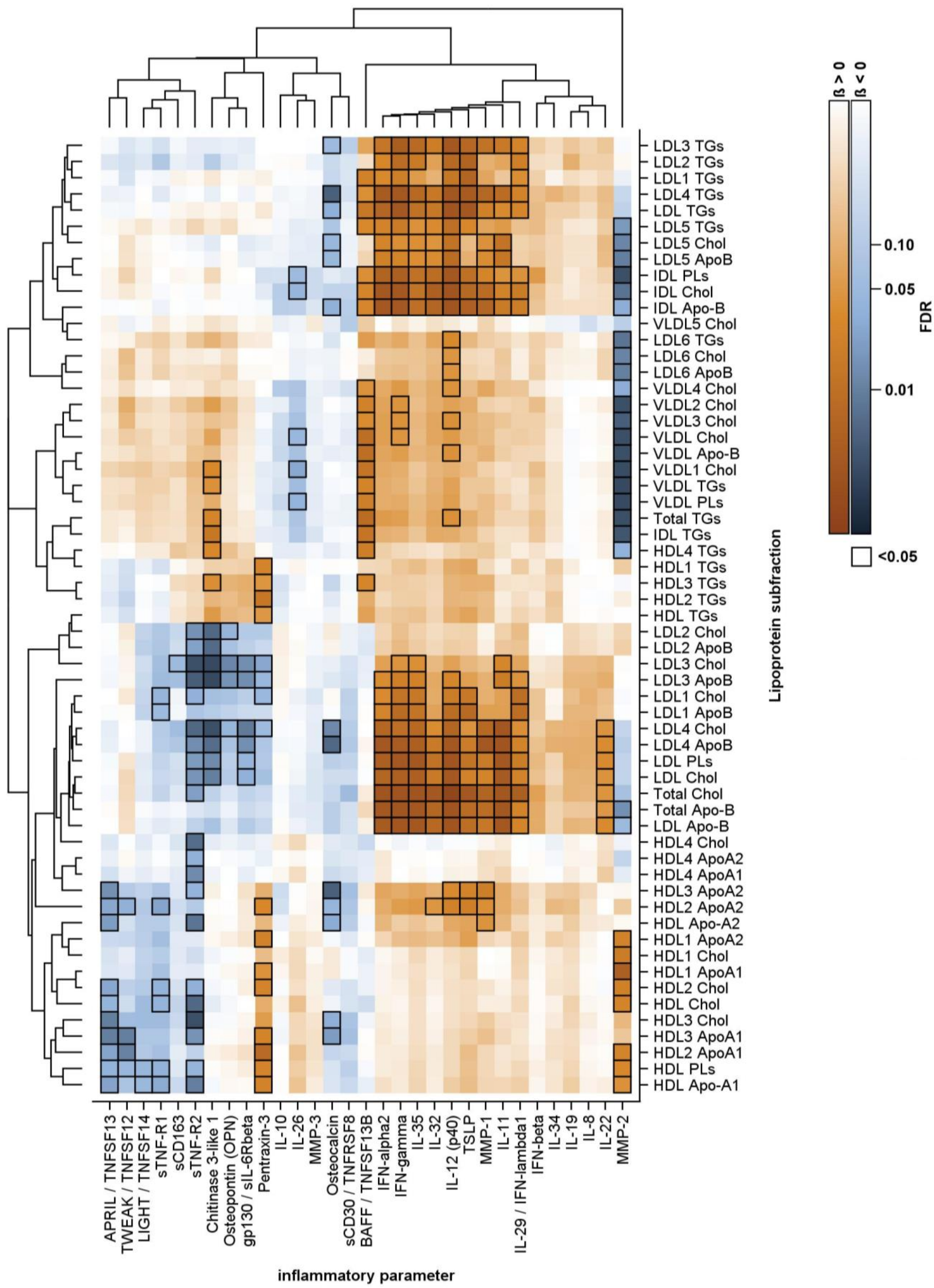

Figure S4. Color coded corrected P values (controlling the false discovery rate (FDR) at 0.05) from linear regression analyses for all investigated associations of lipoprotein subclasses with inflammatory parameters. Significant associations (FDR < 0.05) are marked with a black box. All analyses were adjusted for age, sex, smoking behavior and lean body mass. Orange and blue shading indicate positive and inverse associations, respectively.

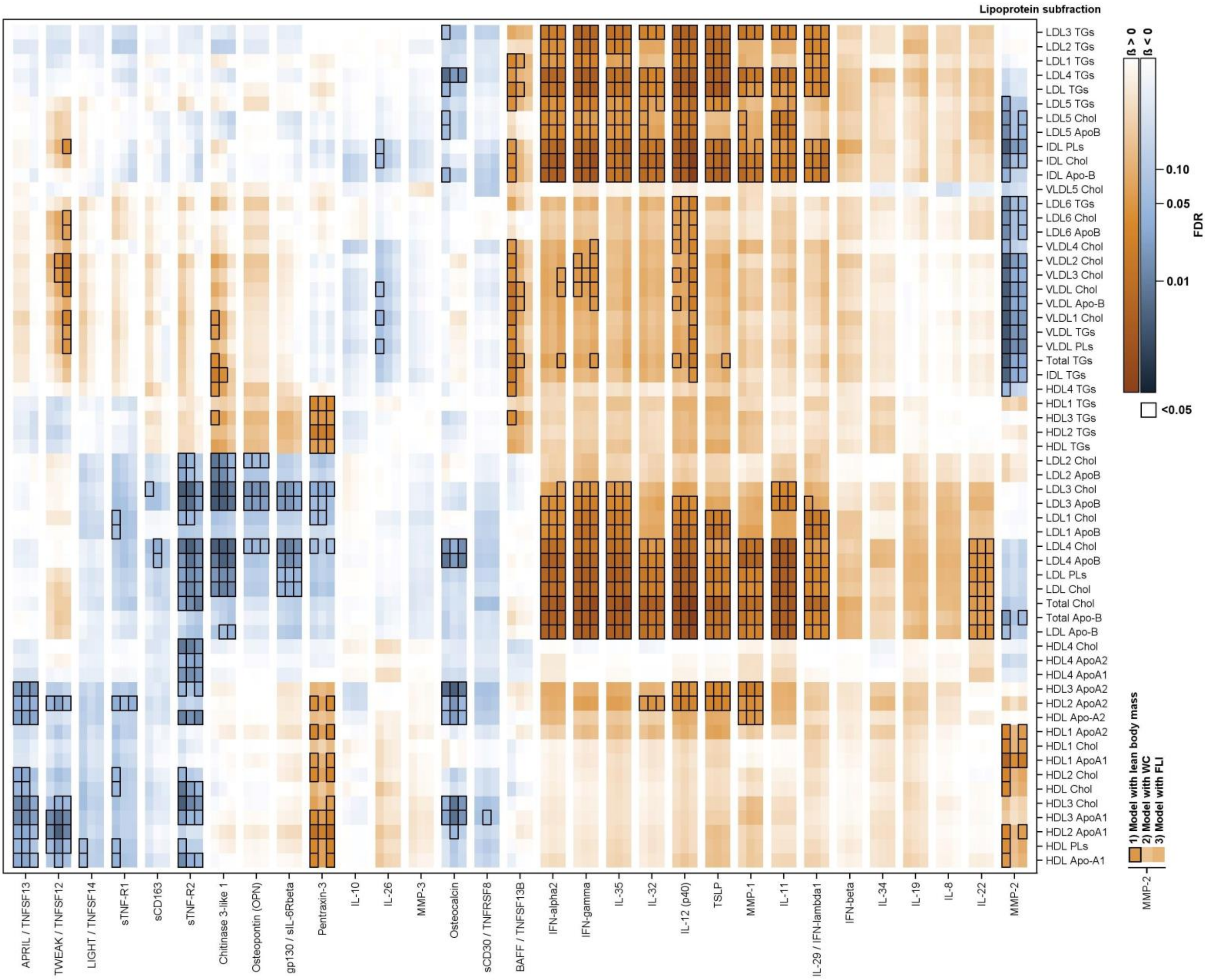

Figure S5. Color coded corrected P values (controlling the false discovery rate (FDR) at 0.05) from linear regression analyses for all investigated associations of lipoprotein subclasses with inflammatory parameters. Significant associations (FDR < 0.05) are marked with a black box. Orange and blue shading indicate positive and inverse associations, respectively. Three different models were calculated:

Model 1) adjusted for age, sex, smoking behavior and lean body mass.

Model 2) adjusted for age, sex, smoking behavior and waist circumference (WC).

Model 3) adjusted for age, sex, smoking behavior and fatty liver index (FLI).
